# CeO_2−δ_ as Electron Donor in Co_0.07_Ce_0.93_O_2−δ_ Solid Solution Boosts Alkaline Water Splitting

**DOI:** 10.1002/advs.202411845

**Published:** 2024-12-02

**Authors:** Gege Su, Yichao Hou, Jie Yin, Jiayi Yang, Zhenglong Li, Xin Du, Xin Zhang, Pinxian Xi, Chun‐Hua Yan

**Affiliations:** ^1^ State Key Laboratory of Applied Organic Chemistry Key Laboratory of Nonferrous Metal Chemistry and Resources Utilization of Gansu Province Frontiers Science Center for Rare Isotopes College of Chemistry and Chemical Engineering Lanzhou University Lanzhou 730000 P. R. China; ^2^ College of Chemistry Zhengzhou University Zhengzhou 450001 China; ^3^ School of Nuclear Science and Technology Lanzhou University Lanzhou 730000 China; ^4^ Beijing National Laboratory for Molecular Sciences State Key Laboratory of Rare Earth Materials Chemistry and Applications PKU‐HKU Joint Laboratory in Rare Earth Materials and Bioinorganic Chemistry College of Chemistry and Molecular Engineering Peking University Beijing 100871 China

**Keywords:** alkaline water splitting, CeO_2−δ_, electronic structures, HER, solid solutions

## Abstract

Optimizing the electronic structure with increasing intrinsic stability is a usual method to enhance the catalysts’ performance. Herein, a series of cerium dioxide (CeO_2−δ_) based solid solution materials is synthesized via substituting Ce atoms with transition metal (Co, Cu, Ni, etc.), in which Co_0.07_Ce_0.93_O_2−δ_ shows optimized band structure because of electron transition in the reaction, namely Co^3+^ (3d^6^4s^0^) + Ce^3+^ (4f^1^5d ^0^6s^0^) → Co^2+^ (3d^7^4s^0^) + Ce^4+^ (4f^0^5d^0^6s^0^), with more stable electronic configuration. The in situ Raman spectra show a stable F2g peak at ≈452 cm^−1^ of Co_0.07_Ce_0.93_O_2−δ_, while the F2g peak in CeO_2−δ_ almost disappeared during HER progress, demonstrating the charge distribution of *H adsorbed on Co_0.07_Ce_0.93_O_2−δ_ is more stable than *H adsorbed on CeO_2−δ_. Density functional theory calculations reveal that Co_0.07_Ce_0.93_O_2−δ_ solid solution increases protonation capacity and favors for formation of *H in alkaline media. General guidelines are formulated for optimizing adsorption capacity and the volcano plot demonstrates the excellent catalytic performance of Co_0.07_Ce_0.93_O_2−δ_ solid solution. The alkaline anion exchange membrane water electrolysis based on Co_0.07_Ce_0.93_O_2−δ_/NiFe LDH realizes a current density of 1000 mA cm^−2^ at ≈1.86 V in alkaline seawater at 80 °C and exhibits long‐term stability for 450 h.

## Graphical abstract

The work introduces a strategy for optimizing the electronic structure of CeO_2_ via substituting Ce atoms with transition metal elements (Cr, Co, Ni, Zn, Cu) to form the solid solution. The Co_0.07_Ce_0.93_O_2‐__δ_ solid solution exhibits an optimized band gap structure and hydrogen adsorption energy, significantly enhancing the activity and stability of water splitting in alkaline media.

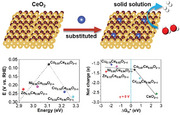

## 1 Introduction

Hydrogen energy is one of the most promising carriers in the future sustainable energy system, and electrocatalytic water splitting for hydrogen production is widely regarded as an innovative solution to energy security challenges.^[^ ^1^, ^2^ ^]^ In this field, catalyst design is key to improving catalytic efficiency and reducing costs. Alkaline anion exchange membrane water electrolysis (AEMWE), based on inexpensive and readily available materials, has been widely adopted as a promising hydrogen production method.^[^ ^3^, ^4^, ^5^, ^6^, ^7^, ^8^ ^]^ Additionally, alkaline seawater electrolysis, an emerging hydrogen production technology, not only utilizes abundant seawater resources but also enhances energy efficiency while lowering costs. Transition metal‐based electrocatalysts, such as layered hydroxides,^[^ ^9^ ^]^ spinel oxides,^[^ ^10^ ^]^ and sulfides,^[^ ^11^ ^]^ typically enhance OER performance more than Ir, Ru, and their corresponding oxides.^[^ ^12^, ^13^ ^]^ However, many of them, like Raney nickel,^[^ ^14^ ^]^ show limited hydrogen production performance in alkaline media due to their restricted protonation ability in alkaline water,^[^ ^15^, ^16^ ^]^ which constrains AEMWE's operation at higher current densities.^[^ ^17^, ^18^ ^]^ This limitation affects their application in anion exchange membrane water electrolysis. Therefore, exploring alternative catalysts with stronger protonation capabilities is crucial for improving the overall performance and efficiency of AEMWE.

Interface engineering,^[^ ^19^ ^]^ atomic exposure,^[^ ^20^ ^]^ and doping strategies^[^ ^21^ ^]^ have demonstrated that HER activity can be optimized through adjustments to electronic structures. Among these, ion doping is one of the effective strategies for enhancing HER performance. Generally, doping with exogenous atoms can alter the unit cell parameters and adjust valence electrons and local coordination, thereby effectively improving the adsorption behavior of reaction intermediates and further optimizing electrocatalytic performance.^[^ ^22^, ^23^, ^24^ ^]^ Cerium dioxide (CeO_2_), as a cheap material with a strong Ce─O bond, exhibits superior stability under an oxygen‐rich atmosphere and the fast redox reaction between Ce^3+^ and Ce^4+^ provides extra electron flow for electrochemical reactions,^[^ ^25^, ^26^, ^27^ ^]^ but high bandgap of CeO_2_ (3.12 eV) limited the intrinsic performance in different electrochemical reactions. Doping transition metals into cerium dioxide can further enhance its catalytic performance. The introduction of exogenous atoms can alter unit cell parameters, adjust valence electrons, and modify local coordination, optimizing the adsorption behavior of reaction intermediates, which is particularly important for the hydrogen evolution reaction (HER). Current research has mostly focused on improving HER performance through noble metal doping, with limited attention on doping with transition metal elements. Although non‐noble metals typically exhibit weaker catalytic performance, their high abundance and low cost still present significant potential for effective catalysis in hydrogen evolution reactions. Therefore, in‐depth studies on the effects of transition metal doping in cerium dioxide could open new avenues and directions for enhancing HER performance. By combining the properties of cerium dioxide with doping strategies involving transition metals, we aim to design more efficient and cost‐effective catalysts, laying the foundation for a sustainable hydrogen energy future.

Herein, a strategy of optimizing CeO_2_ electronic structure was fabricated via transition metal atoms (M: Co, Ni, Zn, Cu, Cr) substituted Ce atoms in CeO_2−δ_ structure and formed M_x_Ce_1−x_O_2−δ_ solid solution materials. Among them, Co_0.07_Ce_0.93_O_2−δ_ with optimized electronic structure shows a slightly decreased bandgap of 3.08 eV and the highest HER performance. Then, a volcano relationship can be found between bandgap and HER activity, in which Co_0.07_Ce_0.93_O_2−δ_ at the top of the volcano means Co is the ideal dopant for CeO_2−δ_ to optimize intrinsic structure and increase the HER activity. The in situ Raman and attenuated total infrared absorption spectroscopy (ATR‐IR) also indicates Co_0.07_Ce_0.93_O_2−δ_ surface with optimized electronic structure is favorable for intermediate adsorption. More importantly, the HER mechanism changed from Volmer–Heyrovsky of CeO_2−δ_ to Volmer–Tafel of Co_0.07_Ce_0.93_O_2−δ_. The density functional theory (DFT) calculations also indicate the optimized *H adsorption (*H_ad_) and promote proton coupling to form hydrogen gas. Interestingly, a volcano curve can be built based on the relationship between Bader charge and ΔG_H__*_ of those solid solution materials, in which Co_0.07_Ce_0.93_O_2−δ_ located at the top of the volcano and closer to *η* = 0 V exhibiting the most excellent HER activity. More importantly, The AEMWE based on Co_0.07_Ce_0.93_O_2−δ_/NiFe LDH can get the current density of 1000 mA cm^−2^ at 2.05 and 1.86 V in alkaline seawater at 25 and 80 °C, respectively.

## 2 Results and Discussion

**Figure 1:**
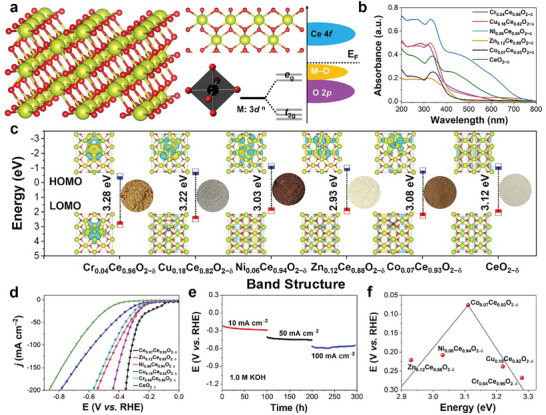
a) Schematic diagrams of the electron orbitals for CeO_2−δ_ and M_x_Ce_1−x_O_2−δ_, illustrating the changes in the orbital configurations. b) UV–vis diffuse reflectance spectra of those materials. c) Band structure diagram and bandgap for those materials with different colors. The insert the calculated HOMO and LUMO of those materials through DFT. d) LSV curves of CeO_2−δ_ and M_x_Ce_1−x_O_2−δ_ for HER. e) Long‐term stability of Co_0.07_Ce_0.93_O_2−δ_ in 1.0 m KOH. f) A volcano plot formed by the combination of *η* and band structure.

The rare earth (RE) elements with unique valence 4*f* ^n−1^5*d* ^0−1^6*s* ^2^ electronic configurations show flexible 4*f* orbital due to the shielding effect of 5*s*/5*p* or 6*s* electrons, suggesting an easy coupling with other orbitals.^[^ ^28^, ^29^, ^30^ ^]^ Ce shows an amazing redox shuttle of Ce^3+^ (4*f* ^1^)−Ce^4+^ (4*f* ^0^) ion redox shuttle because of the unique valence state shift from Ce^3+^ to Ce^4+^ with the formation of a more stable 4*f*° configuration. Therefore, CeO_2−δ_ with reversible charge transfer between Ce^4+^ and Ce^3+^ makes it an ideal model to understand the *d*−*f* electronic interaction in M_x_Ce_1−x_O_2−δ_. In detail, the O_h_−[CeO_6_] unit next to the [MO_6_] unit with a_1g_, t_1u_, e_g_, t_2g_ symmetric orbitals and corresponding antibonding states of a_1g_*, t_1u_*, e_g_*, and t_2g_*, splits the seven degenerate 4*f* orbitals of the Ce atom into t_1u_, t_2u_, and a_2u_ symmetry. Thus, build the orbital coupling and form the 3*d*_M_−2*p*_O_−4*f*_Ce_ in M_x_Ce_1−x_O_2−δ_ with the t_1u_ and t_2u_ symmetric orbitals contribute to the RE─O bonding and the e_g_ and t_2g_ symmetric orbitals correlating with M─O bonding (**Figure** **1**a). Then, we synthesized a series CeO_2−δ_ based solid solution with transition metal ion (Cr, Zn, Co, Cu, Ni) substituted Ce position via hydrothermal method.^[^ ^31^ ^]^ Despite introducing hetero‐metal ions, the powder X‐ray diffraction patterns of as‐synthesized M_x_Ce_1−x_O_2−δ_ (Figure S1, Supporting Information) display a single crystalline phase attributed to the fluorite structure of CeO_2−δ_ (space group #43‐1002, Fm $\bar{3}$ m). Additionally, the Raman spectra of those prepared materials (Figure S2, Supporting Information) also show the main peaks of CeO_2−δ_. Therefore, they could form a substituted M_x_Ce_1−x_O_2−δ_ solid solution because the volume of ions (Cr^3+^: 69 pm, Zn^2+^: 74 pm, Co^2+^: 63 pm, Cu^2+^: 72 pm, Ni^2+^: 62 pm) bigger than the interstitial space of CeO_2_ cell. The inductively coupled plasma optical emission spectroscopy was used to test the content of those metal ions and obtain the chemical formula of those materials (Table S1, Supporting Information). Compared to pure CeO_2−δ_, the metal‐substituted solid solution exhibited a corresponding blue shift or red shift in the absorption edge wavelength in UV–vis spectra (Figure 1b).

The band structure diagrams (Figure 1c) were obtained through UV–vis diffuse reflectance spectroscopy and Mott–Schottky plots (Figure S3, Table S2, Supporting Information).^[^ ^32^ ^]^ Additionally, we analyzed the highest occupied molecular orbital (HOMO) and lowest unoccupied molecular orbital (LUMO) of original CeO_2−δ_ and other solid solution materials through DFT. For the pristine CeO_2−δ_, no obvious molecular orbitals are formed on its surface, but *σ/π* mixing bond formation are formed at the bottom. When Cu, Cr, Co, Ni, and Zn ions replace Ce atoms, the HOMO of CeO_2−δ_ forms *σ/π* mixing molecular orbitals (insert in Figure 1c).^[^ ^33^ ^]^ As we all know, the overlap degree of *π* bond is smaller than that of *σ* bond, so the bond energy of *π* bond is smaller than that of σ bond, its stability is lower than that of *σ* bond, the bond electrons are more active, so it is an active participant in chemical reactions. The HER performance of those materials was evaluated by using the linear scan voltammogram (LSV) in 1.0 m KOH (Figure 1d).^[^ ^34^ ^]^ Among them, Co_0.07_Ce_0.93_O_2−δ_ shows the best HER activity with a *η* of 75.18 mV at *j* = 10 mA cm^−2^ which is much lower than that of CeO_2−δ_ (419.68 mV), Cr_0.04_Ce_0.96_O_2−δ_ (266.89 mV), Cu_0.18_Ce_0.82_O_2−δ_ (233 mV), Zn_0.12_Ce_0.88_O_2−δ_ (221.1 mV), Ni_0.06_Ce_0.94_O_2−δ_ (210.46 mV). More importantly, Co_0.07_Ce_0.93_O_2−δ_ also shows excellent HER stability for 300 h under different current densities (10, 50, and 100 mA cm^−2^) (Figure 1e). Finally, a volcano image can be found based on the HER activity and bandgap of those materials, in which Co_0.07_Ce_0.93_O_2−δ_ solid solution at the top of the volcano.

**Figure 2:**
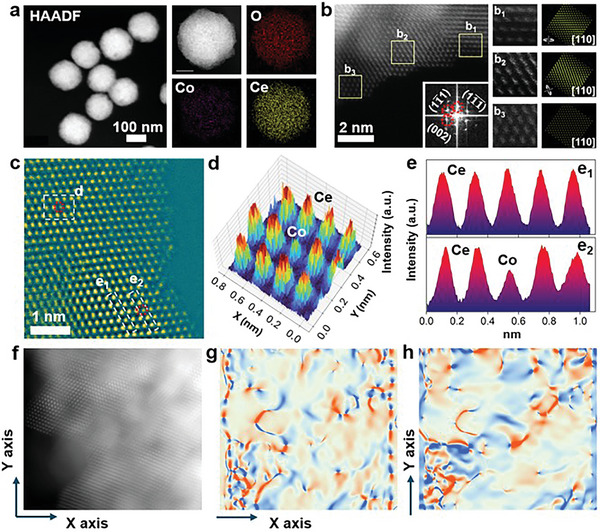
a) STEM, b) HR‐STEM, and c) AC‐STEM of Co_0.07_Ce_0.93_O_2−δ_. The difference between the pure Ce column and the Ce + Co column along the d) 3D intensity and e) line intensity distributions in (c). f) The strain field distribution in g) *X* and h) *Y* directions, with the strain degree from −3% to 3%.

To explain the excellent HER performance of Co_0.07_Ce_0.93_O_2−δ_, the high angle annular dark field‐scanning transmission electron microscopy (HAADF‐STEM) was used to study the atomic structure of Co_0.07_Ce_0.93_O_2−δ_ solid solution. Both CeO_2−δ_ (Figure S4, Supporting Information) and Co_0.07_Ce_0.93_O_2−δ_ (**Figure** **2**a) show the nanosphere morphology in HAADF‐STEM images, demonstrating that Co‐substituting Ce atoms in CeO_2−δ_ structure maintain the nanosphere morphology. The average particle size of Co_0.07_Ce_0.93_O_2−δ_ solid solution was ≈166 nm confirmed by distribution statistics (Figure S5, Supporting Information). The Energy dispersive X‐ray spectroscopy elemental mapping results (**Figure** 2a; Figure S6, Supporting Information) indicate the uniform distribution of Co, Ce, O and Ce, O in the areas of multi‐particles, single particle, and particle surface (as micrometer scale to sub‐nanometer scale). The high‐resolution scanning transmission electron microscopy (HR‐STEM) images reveal the hierarchical structure of nanospheres. CeO_2−δ_ nanospheres are stacked by the sheets with stepped atomic edges (Figure S7, Supporting Information), which will exist abundant edge active sites for catalytic reactions. Furthermore, the substructure of Co_0.07_Ce_0.93_O_2−δ_ also maintains nanosheets atmosphere (**Figure** 2b) with corresponding fast Fourier transform (FFT) face of (11$\bar{1}$), (1$\bar{1}\bar{1}$), (002) and the nanosheets preferers orientation of the {110} crystal plane family for CeO_2−δ_ in a different area (**Figure** 2b_1_–b_3_).

To further investigate the existence form of Co atoms in Co_0.07_Ce_0.93_O_2−δ_, the aberration‐corrected scanning transmission electron microscopy (AC‐STEM) was used (Figure 2c). Usually, the image intensity of HAADF‐STEM is approximately proportional to the sample thickness and the atomic number as Z^1.8^, which is widely used to confirm the metal atoms in different systems. Therefore, both 3D intensity (Figure 2d) and line intensity distributions (Figure 2e) show the contrast difference between the pure Ce column and the Ce + Co column demonstrating Co atoms substituted the Ce position and formed Co_0.07_Ce_0.93_O_2−δ_ solid solution. Based on high‐resolution atomic images, the geometrical phase analysis (GPA) was further used to study the strain change after the formation of Co_0.07_Ce_0.93_O_2−δ_ solid solution. As shown in (Figure 2f–h), the strain field distribution in the *X* and *Y* directions is given with the strain degree from −3% to 3%, indicating the Co substituting Ce atoms and forming Co_0.07_Ce_0.93_O_2−δ_ structure will not bring strain effect in the material.

**Figure 3:**
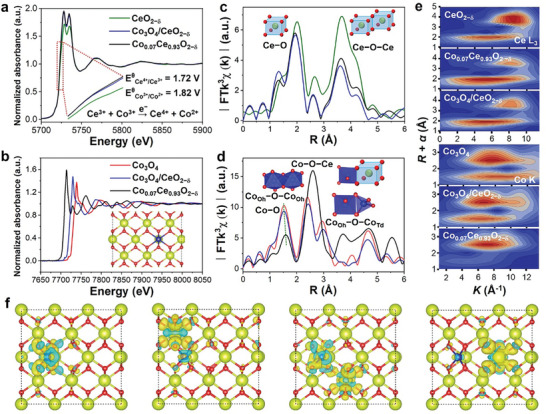
The normalized a) Ce L_3_‐edge, and b) Co K‐edge EXAFS spectra for CeO_2−δ_, Co_3_O_4_, Co_0.07_Ce_0.93_O_2−δ_, and Co_3_O_4_/CeO_2−δ_. c) Ce L_3_‐edge and d) Co K‐edge FT‐EXAFS in R space for CeO_2−δ_, Co_3_O_4_, Co_0.07_Ce_0.93_O_2−δ_, and Co_3_O_4_/CeO_2−δ_. The fitting results for e) WT analysis of Ce L_3_‐edge and Co K‐edge. f) The corresponding charge density differences of Co_0.07_Ce_0.93_O_2−δ_.

The electronic interaction between Co and CeO_2−δ_ spectra.^[^ ^35^, ^36^ ^]^ Compared to CeO_2−δ_, a noticeable enhancement in white line intensity of Ce L_3_‐edge XANES spectra is observed for Co_0.07_Ce_0.93_O_2−δ_ and Co_3_O_4_/CeO_2−δ_ (**Figure** **3**a), implying the strong coupling between two different species. Meanwhile, the shoulder peak at 5724.5 eV in Ce L_3_‐edge XANES spectra attributed to Ce^3+^ decreased after the introduced Co species,^[^ ^37^ ^]^ further demonstrating the CeO_2_ and Co species probably serve as the electron donor and electron receptor in Co_0.07_Ce_0.93_O_2−δ_ structure, respectively. This conclusion can also be revealed by the Co K‐edge XANES spectra (Figure 3b), in which Co_0.07_Ce_0.93_O_2−δ_ shows more negative edge shift compared with that of CeO_2−δ_ and Co_3_O_4_/CeO_2−δ_, indicating the decrease of the Co oxidation state of Co_0.07_Ce_0.93_O_2−δ_ because of the electron transfer from CeO_2_ to Co species. Additionally, the standard electrode potentials (*E^θ^*) of Ce^4+^/Ce^3+^ and Co^3+^/Co^2+^ are 1.72 and 1.82 V, respectively, meaning the reaction between Ce^3+^ and Co^3+^ with an electron‐transition (Ce^3+^ + Co^3+^ → Ce^4+^ + Co^2+^). The formation of the Co_0.07_Ce_0.93_O_2−δ_ structure is further confirmed by Ce L_3_‐edge and Co K‐edge extended X‐ray absorption fine structure (EXAFS) spectra. As shown in the Ce L_3_‐edge EXAFS spectra (Figure 3c), CeO_2−δ_ exhibits two main peaks at ≈2.01 Å for Ce─O and ≈3.65 Å for Ce─O─Ce bond. An obvious negative shift can be found in Ce L_3_‐edge R space of Co_0.07_Ce_0.93_O_2−δ_ and Co_3_O_4_/CeO_2−δ_ with ≈1.95 Å for Ce─O, ≈3.60 Å (Co_3_O_4_/CeO_2−δ_) and ≈3.62 Å (Co_0.07_Ce_0.93_O_2−δ_) for Ce─O─Ce bond. Additionally, Co_0.07_Ce_0.93_O_2−δ_ and Co_3_O_4_/CeO_2−δ_ show a clear peak centered at ≈1.35 Å because of the interaction of Co and Ce between O. The Ce─O─Co bonding interaction can also be revealed by the Co K‐edge EXAFS spectra (Figure 3d). Compared to the Co_3_O_4_ spinel structure, a stronger peak at ≈2.58 Å for the Co─O─Ce structure appeared in Co_0.07_Ce_0.93_O_2−δ_, while the Co_3_O_4_/CeO_2−δ_ heterostructure just shows slightly positive shift for three main peaks. More importantly, combined with the least‐squares EXAFS curve‐fitting analysis (Figure S8, Table S6, Supporting Information), it can be concluded that Co substituted Ce atoms and formed Co_0.07_Ce_0.93_O_2−δ_ solid solution structure. The wavelet transform (WT) analysis also identifies the existence of both Ce─O, Ce─O─Ce, Co─O, and Co─O─Ce bonds (Figure 3e) in Co_0.07_Ce_0.93_O_2−δ_. These above characterizations prove that Co atoms doped into the CeO_2_ lattice can be stabilized and form Co─O_4_ sites in the Co─O─Ce structure. Further, the existence of Co─O─Ce bonding enhanced the first coordination shell of Ce─O, which also contributes to the stability of the Co_0.07_Ce_0.93_O_2−δ_ in electrochemical reactions.

Furthermore, X‐ray photoelectron spectroscopy (XPS) was utilized to investigate the surface chemical states and interface charge transfer of the materials.^[^ ^38^ ^]^ XPS spectra revealed that the predominant peaks of CeO_2−δ_ corresponded to Ce^4+^ 3*d*_5/2_, Ce^4+^ 3*d*_3/2_, Ce^3+^ 3*d*_3/2_, and Ce^3+^ 3*d*_5/2_, with respective binding energy of 919.80, 902.50, 902.10, and 884.20 eV (Figure S9, Tables S3–S5 Supporting Information). Comparatively, the Co_0.07_Ce_0.93_O_2−δ_ solid solution and Co_3_O_4_/CeO_2−δ_ heterostructure exhibited slight shifts in the binding energies of Ce^3+^ 3*d*_5/2_ and 3*d*_3/2_ peaks toward higher values, whereas the binding energies of Ce^4+^ 3*d*_5/2_ and 3*d*_3/2_ peaks displayed a slight shift toward lower values. The Ce^3+^/Ce^4+^ peak area ratio decreased from 0.84 to 0.70 and 0.82, indicating partial oxidation of Ce^3+^ to Ce^4+^ in the presence of Co and the occurrence of charge transfer at the interface, consistent with the XANES data. Compared to CeO_2−δ_ and Co_3_O_4_, the O 1*s* XPS lattice oxygen peak of both Co_0.07_Ce_0.93_O_2−δ_ and Co_3_O_4_/CeO_2−δ_ exhibit positive and negative shifts confirming the electron transfer process between Co and Ce. Furthermore, the binding energy of Co 2*p*₃/₂ in Co_0.07_Ce_0.93_O_2−δ_ and Co_3_O_4_/CeO_2−δ_ shows a negative shift compared to Co_3_O_4_, providing further evidence of electron transfer and electron coupling between Co and Ce. The charge density changes in Co_0.07_Ce_0.93_O_2−δ_ between the surface atoms and the atoms in the next layer (Co and Ce) of CeO_2−δ_ before and after Co substituting were further studied to evaluate the electron transition by DFT. The charge distribution of Ce atoms in pristine CeO_2−δ_ at the four positions is the same with the electrons of Ce and O transfer to the middle position (Figure S10, Supporting Information). However, the stronger bonding of Ce─O in the Co_0.07_Ce_0.93_O_2−δ_ solid solution can be found (Figure 3f). Then, we analyzed the differential charge of the two atoms of Co_0.07_Ce_0.93_O_2−δ_ and pristine CeO_2−δ_ (Figure S11, Supporting Information). The charge distribution around the two Ce atoms and O atoms on the surface of the original CeO_2−δ_ is uniform, and there is no charge offset phenomenon. However, when Co atoms replace Ce atoms, the charge distribution is uneven. The electrons around Ce and O atoms have a slight tendency to shift toward Co, which further illustrated that the Ce atoms could provide electrons to Co atoms in Co_0.07_Ce_0.93_O_2−δ_ solid solution.

Then, The HER performance of CeO_2−δ_, Co_0.07_Ce_0.93_O_2−δ_ solid solution, Co_3_O_4_/CeO_2−δ_ heterostructure, and Co_3_O_4_ was further evaluated in alkaline media. As expected, Co_0.07_Ce_0.93_O_2−δ_ displays the best HER performance with the lowest overpotential (75.18 mV) than that of CeO_2−δ_ (419.68 mV), Co_3_O_4_/CeO_2−δ_ (280.45 mV), and Co_3_O_4_ (148.60 mV) at *j* = 10 mA cm^−2^ (Figure S12, Supporting Information). Meanwhile, Co_0.07_Ce_0.93_O_2−δ_ annealing under 500 °C shows the best HER performance compared with the materials obtained at 300 and 700 °C (Figure S13, Supporting Information). By comparing the cyclic voltammetry, change‐data‐capture listing (*C*_dl_), and electrochemical impedance spectroscopy of CeO_2−δ_ and Co_0.07_Ce_0.93_O_2−δ_, it can be more intuitively observed that Co_0.07_Ce_0.93_O_2−δ_ has more excellent electrochemical properties (Figures S14–S16, Supporting Information). Therefore, Co_0.07_Ce_0.93_O_2−δ_ solid solution with 3*d*_Co_−2*p*_O_−4*f*_Ce_ orbital coupling optimized electronic structure shows excellent HER performance compared with other recently reported works (Table S7, Supporting Information).

**Figure 4:**
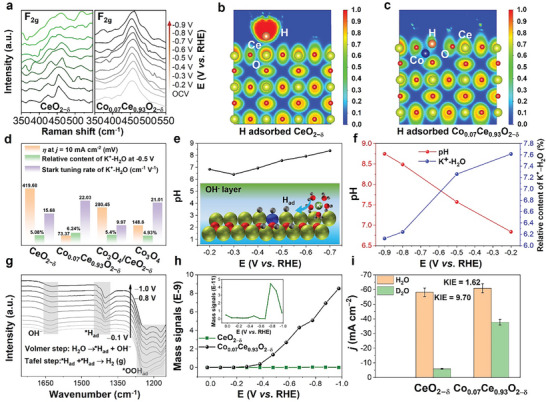
a) The in situ Raman spectra of CeO_2−δ_ and Co_0.07_Ce_0.93_O_2−δ_. b,c) The ELF of H adsorption on CeO_2−δ_ and Co_0.07_Ce_0.93_O_2−δ_. d) The influence of interfacial water on alkaline HER performance. e) Local pH of Co_0.07_Ce_0.93_O_2−δ_ in alkaline HER process. f) The Relationship between local pH and K^+^·H_2_O. g) The in situ ATR‐IR spectra of Co_0.07_Ce_0.93_O_2−δ_ for HER. h) The DEMS signals of CeO_2−δ_ and Co_0.07_Ce_0.93_O_2−δ_. i) The KIE of CeO_2−δ_ and Co_0.07_Ce_0.93_O_2−δ_.

Furthermore, the in situ spectra were used to confirm the catalysts’ structure and adsorption intermedia in HER. First, the in situ Raman spectra were used to study the structure of those catalysts in HER. As shown in **Figure** **4**a, the characteristic vibrational peak of CeO_2−δ_ at ≈452 cm^−1^ for the *F*_2g_ peak decreased because the charge distribution in the Ce−O−Ce structure changed with enhanced *H_ad_ under increased HER potentials. Surprisingly, the *F*_2g_ peak for CeO_2_ in Co_0.07_Ce_0.93_O_2−δ_ structure maintains under the same conditions demonstrating the Co substituting Ce atomic position in CeO_2−δ_ structure can further stabilize the charge structure under HER.^[^ ^39^ ^]^ Then, the DFT calculation also evidenced the H adsorption processes of CeO_2−δ_ and Co_0.07_Ce_0.93_O_2−δ_. The results of electronic localization function (ELF) for pristine CeO_2−δ_ and Co_0.07_Ce_0.93_O_2−δ_ adsorption of H ions (Figure 4b,c). The maximum ELF value between Co and H is ≈0.6, indicating a weak covalent‐like nature of the Co─H bond,^[^ ^40^ ^]^ while the Ce─H bond has a larger maximum ELF value demonstrating a stronger adsorption capacity for H on the pristine CeO_2−δ_, which is very unfavorable for the desorption of H. This in turn confirms that Co doping could effectively reduce the adsorption capacity of the overall material for H and could effectively improve the HER performance. Additionally, in situ Raman spectra from 3000 to 3700 cm^−1^ of those catalysts (Figures S17,S18, Supporting Information) are assigned to the O–H stretching mode (ν_O–H_) of interfacial water with three distinct components of interfacial water, namely 4−HB·H_2_O, 2−HB·H_2_O, and K^+^·H_2_O, respectively.^[^ ^41^ ^]^ The frequencies of adsorbate exhibit the vibrational Stark effect, and the steeper Stark tuning rate shows high sensitivity to the local environment of the electrode. In detail, Co_0.07_Ce_0.93_O_2−δ_ shows higher content of K^+^·H_2_O and Stark tuning rate of (22.03 cm^−1^ V^−1^) than those of CeO_2−δ_, Co_3_O_4,_ and Co_3_O_4_/CeO_2−δ_ (Figure 4d), demonstrating more free water on Co_0.07_Ce_0.93_O_2−δ_ interface. Therefore, the Co_0.07_Ce_0.93_O_2−δ_ solid solution structure optimized adsorbed K^+^·H_2_O and reduced the OH^−^ ions coverage on the interfacial water layer, promoting HER processes because active sites can adsorb more proton from free water molecules, which is consistent with the Volmer step (H_2_O + e^−^ → *H_ad_ + OH^−^). The RRDE method was used to further study the relationship between interfacial free water and local pH on the Co_0.07_Ce_0.93_O_2−δ_ surface.^[^ ^42^ ^]^ Based on the negative correlation between local pH variation and Raman interface water (Figure 4e), namely the content of interface water decreases as the local pH increases, indicating that the presence of interface water is suppressed under high pH conditions. This negative correlation can be explained through the following mechanistic analysis. In a high pH environment, the concentration of hydroxide ions (OH⁻) is higher. These hydroxide ions react with hydrogen ions (H⁺) in the interface water, leading to the dissociation of water molecules and a decrease in free water content. Additionally, under high pH conditions, hydroxide ions may also react with other substances at the interface, forming hydrates or other compounds. These reactions may result in the transformation or depletion of interface water, thereby reducing the content of interface water. Therefore, based on the negative correlation between local pH variation and interface water, we can conclude that an increase in local pH under high pH conditions leads to a decrease in interface water (Figure 4f). This finding reveals the influence of local pH on the dynamic equilibrium of interface water, providing important clues for a deeper understanding of interfacial reactions and phenomena.

The in situ ATR‐IR measurement further demonstrated the adsorption intermedia in HER.^[^ ^4^ ^]^ The in situ ATR‐IR spectra of Co_0.07_Ce_0.93_O_2−δ_ show obvious *OOH adsorption (*OOH_ad_) intermedia peaks with increased HER potentials (Figure 4g), which can be attributed to the first step, namely water dissociation (Volmer step: H_2_O + e^−^ → *H_ad_ + OH^−^), for HER in alkaline media. The enhanced *H_ad_ peaks also confirmed the second step for HER, namely the Tafel step (*H_ad_ + *H_ad_ → H_2_(g)), with a faster HER rate than that of CeO_2−δ_ under the same condition (Figure S19, Supporting Information). The in situ isotope‐labeled differential electrochemical mass spectrometry (DEMS) tests were conducted in 1.0 m KOH‐H_2_O and KOH‐D_2_O solutions (Figures S20,S21, Supporting Information). It can be observed that CeO_2‐δ_ and Co_3_O_4_/CeO_2−δ_ exhibit a decrease or leveling off of hydrogen with increased potentials due to the highly enhanced adsorption of *H on CeO_2−δ_ at high HER potentials (Figure S20, Supporting Information). Except for Co_0.07_Ce_0.93_O_2−δ_, _the_ other three materials produce similar amounts of HD and DD in KOH‐D_2_O solution, indicating the occurrence of both HD generation (H^+^/D^+^ exchange) and DD generation reactions in D_2_O both co‐existing (Figure S21, Supporting Information). This phenomenon suggests Co_0.07_Ce_0.93_O_2−δ_ exhibits good HER activity in a wide potential window, and the rate of HER accelerates with higher overpotentials. The kinetic isotope effect (KIE) test was conducted (Figure 4i), and the KIE of CeO_2−δ_ is 9.70 exhibiting a significantly higher value than 1, indicating the Volmer step is the rate‐determining step. Combined with DEMS, a substantial amount of HD gas was generated, suggesting the occurrence of H/D exchange and involvement of the Heyrovsky step during HER with the Volmer–Heyrovsky mechanism. However, the KIE of Co_0.07_Ce_0.93_O_2−δ_ is 1.62 exhibiting a value close to 1, indicating that the proton transfer step is not the rate‐determining step. The substantial production of DD indicates that the Tafel step dominates the reaction and primarily follows the Volmer–Tafel mechanism.^[^ ^43^ ^]^ Therefore, Co substituting Ce in CeO_2−δ_ structure alters the HER mechanism of pure CeO_2−δ_, which can be attributed to the enhanced proton coupling on Co_0.07_Ce_0.93_O_2−δ_ with the improved catalytic activity of HER. Additionally, the structure and morphology of Co_0.07_Ce_0.93_O_2−δ_ were maintained after long‐term stability tests (Figure S22, Supporting Information).

**Figure 5:**
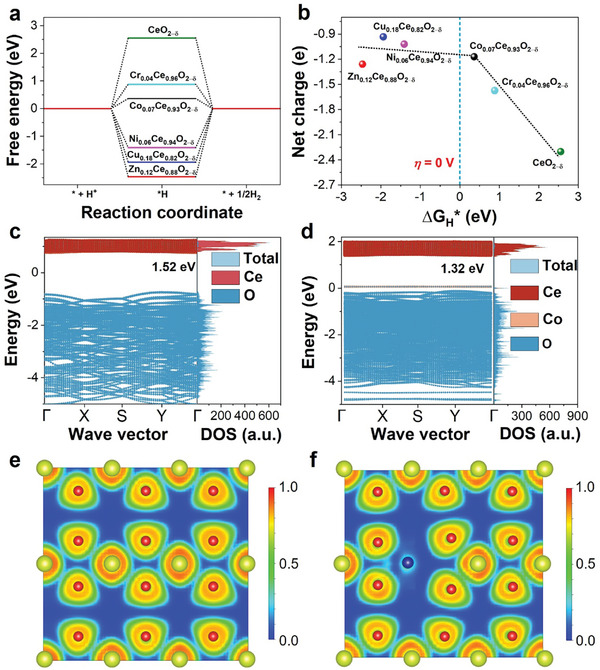
a) The calculated Gibbs free energy diagram (∆G_H__*_) for the hydrogen adsorption on the pristine CeO_2−δ_ and M_x_Ce_1−x_O_2−δ_. b) The relationship between Bader net charge and ∆G_H__*_ of those materials. c,d) DOS and Band, and e,f) ELF of CeO_2−δ_ and Co_0.07_Ce_0.93_O_2−δ_.

The DFT methods were used to calculate the hydrogen adsorption Gibbs free energy (ΔG_H__*_) and evaluate the HER activity by comparing the value of |ΔG_H__*_| on different materials. The calculated ΔG_H__*_ on the CeO_2−δ_ is ≈2.55 eV (**Figure** **5**a), which is higher than that of other solid solution catalysts. Notably, ΔG_H__*_ of Co_0.07_Ce_0.93_O_2−δ_ is only 0.36 eV, which is distinctly lower than Cr_0.04_Ce_0.96_O_2−δ_ (0.88 eV), Cu_0.18_Ce_0.82_O_2−δ_ (1.94 eV), Ni_0.06_Ce_0.94_O_2−δ_ (1.41 eV), and Zn_0.12_Ce_0.88_O_2−δ_ (2.47 eV), demonstrating transition metal substituting promotes H adsorption and desorption behaviors in HER. Interestingly, a volcano curve can be obtained based on the function between Bader net charge value (Figure S23, Supporting Information) and ΔG_H__*_ of pristine CeO_2−δ_ and M_x_Ce_1−x_O_2−δ_ for HER (Figure 5b), the negative sign represents the loss of charge, in which Co_0.07_Ce_0.93_O_2−δ_ locates in the middle of the volcano curve and closest to the *η* = 0 V position, indicating that Co_0.07_Ce_0.93_O_2−δ_ has the most excellent HER catalytic performance. Then, the density of states (DOS, TDOS/PDOS) and Band of those materials were calculated with the Fermi level aligned at 0 eV. Compared to CeO_2−δ_, Co_0.07_Ce_0.93_O_2−δ_ shows a decreased band gap, and an impurity energy level composed of Co–O is generated between the conduction band and the valence band (VB) (Figure 5c,d), which is beneficial to the transfer of electrons and significantly improve the HER properties.^[^ ^44^ ^]^ The intensity of band and DOS on the Co_0.07_Ce_0.93_O_2−δ_ near the VB increased compared to CeO_2−δ_, suggesting conductivity increased after Co substituting Ce position. This phenomenon is also found in the DOS and band results of other solid solutions (Figure S24, Supporting Information) with increased conductivity. The ELF and 2D‐charge density cross‐section of those materials were further studied. The electron localization is reduced after Co, Cr, Cu, Ni, and Zn substituting Ce position in CeO_2−δ_ structure (Figure 5e,f; Figure S25, Supporting Information), indicating transition metal can effectively reduce the binding ability of atomic nuclei to electrons and enhance their mobility.^[^ ^45^ ^]^ This is very beneficial for the transfer of electrons in the catalytic process. We also found that the electron center shifted to the doping position, which further proved Ce can donate electrons to Co. The charge density of pristine CeO_2−δ_ is evenly distributed, while the charge density of Ce near Co in Co_0.07_Ce_0.93_O_2−δ_ is significantly higher (Figure S26, Supporting Information), which further illustrates that the charge shift is consistent with the trend of ELF. Similarly, we also found that since the substituting atoms bind electrons less strongly than Ce atoms, the charge density around them is reduced.

**Figure 6:**
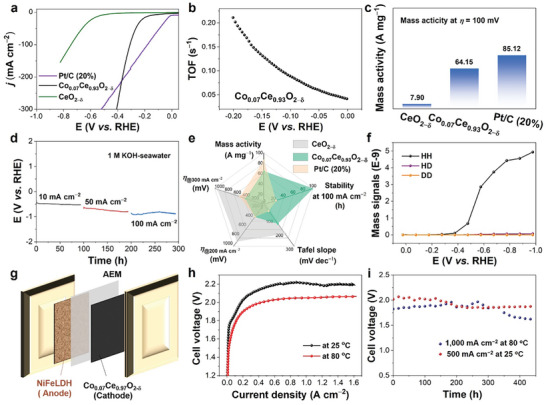
a) The LSV polarization curves of CeO_2−δ_, Co_0.07_Ce_0.93_O_2−δ_ and Pt/C (20%). b) TOF, and c) mass activity of those materials. d) The long‐term stability of Co_0.07_Ce_0.93_O_2−δ_ in 1.0 m alkaline seawater. e) The Radar chart of CeO_2−δ_, Co_0.07_Ce_0.93_O_2−δ_ and Pt/C (20%). f) The DEMS signals of Co_0.07_Ce_0.93_O_2−δ_ in 1.0 m alkaline seawater. g) The diagram of the membrane electrode schematic. h) LSV, and i) stability of Co_0.07_Ce_0.93_O_2−δ_ and NiFe LDH fabricated device for seawater splitting in alkaline media.

We move on to the performance of Co_0.07_Ce_0.93_O_2−δ_ for both actual and alkaline seawater splitting (Figures S27,S28, Supporting Information). The catalytic performance for HER was evaluated by using the LSV in alkaline seawater (1.0 m KOH). In an alkaline seawater system, compared with commercial Pt/C (20%), Co_0.07_Ce_0.93_O_2−δ_ shows lower *η* at higher current density (larger than 200 mA cm^−2^), but CeO_2‐δ_shows negligible HER activity under the same condition (**Figure** **6**a). The turnover frequencies (TOF), responding to the intrinsic activity per site, are calculated based on the hypothesis all Co─O─Ce active centers can access the reaction. The TOF of Co_0.07_Ce_0.93_O_2−δ_ is 0.21 s^−1^ at *η* of 0.2 V (Figure 6b). Meanwhile, Co_0.07_Ce_0.93_O_2−δ_ also shows higher mass activity of 64.15 A mg^−1^ at 100 mV closer to that of Pt/C (20%) (Figure 6c). Additionally, Co_0.07_Ce_0.93_O_2−δ_ displays excellent HER stability for 300 h under different current densities (10, 50, and 100 mA cm^−2^) in alkaline seawater (Figure 6d), while Pt/C (20%) only reaches 25 h (Figure S29, Supporting Information), suggesting Co_0.07_Ce_0.93_O_2−δ_ has superior durability at high current densities in alkaline seawater splitting. The electrocatalytic performances of CeO_2−δ_, Co_0.07_Ce_0.93_O_2−δ_, and Pt/C (20%) were further evaluated and compared using a radar chart analysis (Figure 6e) and other recently reported works (Table S8, Supporting Information), demonstrating Co_0.07_Ce_0.93_O_2−δ_ is a promising catalyst for alkaline seawater splitting. Moreover, the DEMS measures further indicate that H_2_ is the only product without producing any other things in Co_0.07_Ce_0.93_O_2−δ_ catalyzed alkaline seawater HER (Figure 6f).

Finally, the AEMWE device was further developed based on Co_0.07_Ce_0.93_O_2−δ_ as cathode and nickel‐iron‐based layered double‐metal hydroxide (NiFe LDH) as the anode (Figure 6g).^[^ ^46^ ^]^ Before testing, the membrane electrode assembly (MEA) was activated by alkaline seawater flow for 20 min to ensure adequate membrane hydration. At 25 °C, the MEA constructed with the optimized Co_0.07_Ce_0.93_O_2−δ_ achieved the current density of 1000 mA cm^−2^ at a cell voltage of 2.05 V. Impressively, when increasing the temperature to 80 °C, the cell voltage significantly decreased to 1.86 V to get the same current density (Figure 6h), demonstrating the excellent high‐temperature activity of this catalyst. In addition, the Co_0.07_Ce_0.93_O_2−δ_ based alkaline seawater splitting device shows excellent long‐term stability for 450 h at 500 and 1000 mA cm^−2^ with a cell voltage of ≈1.89 and ≈1.75 V at 25 and 80 °C (Figure 6i), indicating its outstanding durability under the operating conditions evaluated.

## Conflict of Interest

The authors declare no conflict of interest.

## Supporting information



Supporting Information

## Data Availability

The data that support the findings of this study are available in the supplementary material of this article.
