# Supplementary material for: CeO2−δ as Electron Donor in Co0.07Ce0.93O2−δ Solid Solution Boosts Alkaline Water Splitting
Source: Adv Sci (Weinh). 2024 Dec 2;12(4):2411845. doi: 10.1002/advs.202411845 (PMC11775571; doi:10.1002/advs.202411845)
Supplement: Supplementary file 1 — Supporting Information [file ADVS-12-2411845-s001.docx]

Supporting Information

**CeO_2−δ_ as Electron Donor in Co_0.07_Ce_0.93_O_2−δ_ Solid Solution Boosts Alkaline Water Splitting**

*Gege Su**^+^, Yichao Hou^+^, Jie Yin^+,^*, Jiayi Yang, Zhenglong Li, Xin Du*, Xin Zhang, Pinxian Xi*, and Chun-Hua Yan*

G. Su, Y. Hou, J. Yin, J. Yang, Z. Li, P. Xi, C.-H. Yan

State Key Laboratory of Applied Organic Chemistry, Key Laboratory of Nonferrous Metal Chemistry and Resources Utilization of Gansu Province, Frontiers Science Center for Rare Isotopes, College of Chemistry and Chemical Engineering, Lanzhou University, Lanzhou, 730000, P. R. China

Email: [yinj@lzu.edu.cn](mailto:yinj@lzu.edu.cn); [xipx@lzu.edu.cn](mailto:xipx@lzu.edu.cn)

X. Du

College of Chemistry, Zhengzhou University, Zhengzhou 450001, China.

X. Zhang

School of Nuclear Science and Technology, Lanzhou University, Lanzhou 730000, China.

C.-H. Yan

Beijing National Laboratory for Molecular Sciences, State Key Laboratory of Rare Earth Materials Chemistry and Applications, PKU-HKU Joint Laboratory in Rare Earth Materials and Bioinorganic Chemistry, College of Chemistry and Molecular Engineering, Peking University, Beijing, 100871, P. R. China

[^+^] These authors contributed equally to this work.

**Experimental section**

**Chemicals and materials:**

**Materials:** Co(NO_3_)_2_·6H_2_O (99%), Ni(NO_3_)_2_·6H_2_O (99%), Zn(NO_3_)_2_·6H_2_O (99%), Cu(NO_3_)_2_·3H_2_O (99%), and Cr(NO_3_)_3_·9H_2_O (99%), Ce(NO_3_)_3_·6H_2_O (99%), Polyvinylpyrrolidone, Ethylene glycol (98%), C_2_H_6_O (98%), and Nickel mesh were purchased from Sigma-Aldrich and used directly without further purification. DI water was supplied by a Millipore system (MilliporeQ).

**Synthesis of CeO_2−δ_:** Weighing 2.0 g of Ce(NO_3_)_3_·6H_2_O and 0.6 g of PVP and dissolve them completely in 75 mL of ethylene glycol and 5 mL of deionized water using a magnetic stirrer. Stir the mixture thoroughly for 30 minutes. The well-mixed solution was transferred to a reaction vessel lined with a 100 mL inner liner and placed in a forced-air drying oven at 180 °C for continuous heating for 8 hours. The cooled sample was subjected to several washes with H_2_O and EtOH by centrifugation, followed by overnight drying in a vacuum oven at 60 °C. Subsequently, the sample was placed in a muffle furnace and calcined for 2 hours at 500 °C (ramp rate 10 °C min^−1^) to obtain the CeO_2−δ_ sample.

**Synthesis of** **M_x_Ce_1_**_−_**_x_O_2_**_−_**_δ_**: Accurately weighing 0.3 g of Co(NO_3_)_2_·6H_2_O, Ni(NO_3_)_2_·6H_2_O, Zn(NO_3_)_2_·6H_2_O, Cu(NO_3_)_2_·3H_2_O, and Cr(NO_3_)_3_·9H_2_O, respectively, they were completely dissolved in a mixture of 75 mL of ethylene glycol and 5 mL of ultrapure water. The subsequent steps are the same as for CeO_2−δ_ synthesis.

**Synthesis of Co_x_Ce_y_O_2_**_−_**_δ_**: The subsequent steps are the same as for CeO_2−δ_, with the addition of 0.1 g, 0.3 g, 2.0 g and 3.0 g of Co(NO_3_)_3_·6H_2_O, respectively.

**Synthesis of Co_3_O_4_:** A total of 72.76 mg of Co(NO_3_)_2_•6H_2_O and 8 mL of glycerol were accurately weighed and completely dissolved in 30 mL of isopropanol under continuous stirring for 30 minutes. The well-mixed solution was transferred to a 100 mL reaction vessel and subjected to continuous reaction at 180 °C for 8 hours. The cooled product was washed several times with H_2_O and EtOH using a centrifuge, followed by overnight drying in a vacuum oven at 60 °C.

**Synthesis of NiFeLDH:** Prepare 0.1 M solutions of Fe(NO₃)₃ and Ni(NO₃)₂, and mix them uniformly in a 1:1 volume ratio to create the mixed electrolyte. In the three-electrode electrochemical deposition setup, a platinum sheet is used as the working electrode, and a silver/silver chloride electrode serves as the reference electrode. Throughout the electro-deposition process, a constant potential of -1 V is applied to the mixed electrolyte, with the deposition time set to 1 hour. After deposition, thoroughly rinse the working electrode with ethanol to remove any residual electrolyte. Then, place the electrode in a vacuum oven at 60 ^o^C and dry it for 2 hours to eliminate ethanol and moisture.

**Sample preparation process for ICP：**Weigh 3 mg of the sample and place it in a 20 mL glass bottle. Add 3 mL of H_2_O_2_ solution to dissolve CeO_2_. Once gas evolution has ceased, add 1 mL of aqua regia under conditions of 90 ^o^C, and heat until the aqua regia has completely evaporated. Stop heating when there is a residual amount of aqua regia, remove the glass bottle, and allow it to cool naturally to room temperature. Then adjust the pH of the solution to 1, transfer it to a 100 mL volumetric flask, and dilute to the mark with deionized water. Take 10 mL of the solution for subsequent analysis.

**Catalyst Characterization:** X-ray diffraction (XRD) results were obtained using a Rigaku MiniFlex 600 diffractometer, operating at a voltage of 40 kV and current of 40 mA, with Cu Ka radiation (λ = 0.1542 nm), scanning from 10° to 90°. Transmission electron microscopy (TEM) and high-resolution transmission electron microscopy (HRTEM) images were employed for atomic-level image analysis using a JEOL JEM 2100 TEM operated at an accelerating voltage of 200 kV. Under the following conditions, transmission electron microscopy (TEM) and high-resolution transmission electron microscopy (HRTEM) images were obtained. The JEOL JEM 2100 TEM was operated at an accelerating voltage of 200 kV. Atomic-level scanning transmission electron microscopy (STEM) images were recorded using a probe aberration-corrected STEM (Cubed Titan G260-300, FEI, USA) operated at 300 kV. X-ray photoelectron spectroscopy (XPS) analysis was performed using a VG ESCALAB 2201-XL instrument. The AIIXPS spectrum was calibrated using the C1s line at 284.8 eV. The sample composition was determined using inductively coupled plasma optical emission spectroscopy (ICP-OES) with a HITACHI P-4010 instrument. Field emission scanning electron microscopy (FESEM) was conducted using a Zeiss instrument at an acceleration voltage of 5 kV. Transmission X-ray absorption spectroscopy (XAS) measurements were conducted on a laboratory instrument (easyXAFS300, easy XAFS LLC) utilizing a Roland geometry setup equipped with a spherical bent crystal analyzer (SBCA) and a silicon drift detector. Silicon was employed for K-edge measurements of Zn, Ni, Cu, Cr, and Co. Powdered samples were thoroughly ground and mixed with boron nitride using a mortar and pestle, followed by compression into 10 mm pellets. The compressed pellets were then sandwiched between Kapton tapes.

**Electrochemical testing HER:** The alkaline hydrogen evolution reaction (HER) tests were conducted on the Corrtest electrochemical workstation (For Corrtest CS series Potentiostat/Galvanostat). Prior to the tests, calibration was calibrated using a standard hydrogen electrode as the reference electrode. The reference electrode used was the alkaline electrode Hg/HgO (1.0 M KOH), and the auxiliary electrode was graphite. The M_x_Ce_1−x_O_2−δ_ was directly used as the working electrode (with a geometric area of 1 cm^2^) to form a three-electrode system. The potential conversion was calculated using the following formula: E_RHE_ = E_(Hg/HgO)_ + 0.098 + 0.0591 pH. All tests were conducted at room temperature (25 ± 0.2 ^o^C)

**In-situ TR-FTIR measurements:** The in-situ TR-FTIR measurements were performed on a Bruker instrument equipped with a DTGS detector. Specifically, a Hg/HgO electrode and a carbon rod were used as the reference electrode and counter electrode, respectively. As for the working electrode, a gold film was deposited on a silicon substrate, followed by the application of a catalyst ink onto the silicon pad surface. During the testing process, a series of i-t measurements were conducted based on continuous potential control. 100 mL 1M KOH was used as the electrolyte, and ATR-FTIR data were obtained after each i-t scan.

**In-situ Raman measurements:** The results of in-situ Raman spectroscopy were obtained using the Horiba LabRAM HR system. The laser wavelength used was 532 nm. Specifically, a Hg/HgO electrode and a carbon rod were employed as the reference electrode and counter electrode, respectively. The catalyst was directly connected to the working station as the working electrode using a copper strip.

**In-situ DEMS measurements:** The DEMS data results were provided by a QAS 100 device (Linglu instruments, Shanghai). The working electrode was prepared by drop-casting onto a polytetrafluoroethylene (PTFE) film surface coated with a layer of Au. The catalyst loading for drop-casting was 0.05 mg cm^−2^. Platinum wire was used as the counter electrode, and a saturated Hg/HgO electrode served as the reference electrode. Subsequently, chronoamperometry (i-t) tests were conducted in the potential range of 0 to −1 V vs. RHE. The catalyst was labeled by preparing a 1.0 M KOD solution in D_2_O, enabling real-time detection of the evolved gas. Mass signals corresponding to hydrogen with different molecular weights were observed.

**Theoretical calculations:** The first-principles density functional theory (DFT) calculations were performed within the generalized gradient approximation (GGA) based on via Vienna abinitio simulation package (VASP)^[1−3]^. The VASP was employed to perform all DFT calculations within the hybrid functional as proposed by Perdew-Burke-Ernzerhof (PBE)^[3,4]^ interactions are represented using the projector augmented wave (PAW)^[3,5,6]^ potential. We constructed CeO_2__−δ_ (110) and M_x_Ce_1−x_O_2−δ_ (110) surface structures, the surface model with periodicity in the x and y directions and the depth of the vacuum layer is greater than 15 Å to prevent self-interactions. In addition, the bottom three stoichiometric layers needed to be fixed while the top layer and adsorbates (H*, H_2_O*, H−OH*) were allowed to relax during the structural optimization process. The Kohn-Sham one-electron valence states were expanded on the basis of plane waves with a cutoff energy of 400 eV in the process of structure optimization. To take care of the strong on-site Coulomb interactions of localized electrons in transition metals, the DFT + U method is used in this study^[7−9]^. The effective U values of 4.5, 3.4, 3.0, 3.0, 3.0, and 8.0 eV were introduced to account for the strong onsite Coulomb repulsion of Ce, Co, Cr, Cu, Ni, and Zn atoms, respectively. Geometry optimization was considered convergent when the electronic energy and Hellmann-Feynman forces convergence criterion was smaller than 10^−5^ eV and 0.03 eV Å^−1^, respectively. The K-point of 2 × 2 × 1 was used for the optimization of all surfaces, and the corresponding adsorption structure (H*, H_2_O*, H−OH*). Subsequently, we calculated the charge density and electrostatic potential in the self-consistent process. The electronic energy was considered self-consistent while the energy variation was smaller than 10^−5^ eV. The value of K-point, cutoff energy is the same as the process of structure optimization. After the self-consistent calculation converges, the charge density distribution and density of states can be obtained, the self-consistent structure is used as the input file, and the wave function and charge density file are read to obtain the density of states.

The Gibbs free energy also was calculated in these studies. The zero-point energy (ZPE) correction was performed referring to the approaches previously reported. In the DFT process, we calculated the Gibbs free energy according to the equations as follows:

$G^{0}=E_{DFT}+ZPE-TS^{0}$ Where *G*^0^ is the Gibbs free energy, E_DFT_ is total free energy, ZPE is the vibration energy; *TS*^0^ is the entropy change (T = 298.15 K). The free energy of adsorption/desorption of H (ΔG_H_*) was defined as:

$$\Delta G_{H}^{*}=G_{H}^{*}-G^{*}-\frac{G_{H_{2}}^{*}}{2}$$

where G_H*_, G* and G_H2_* are the free energy of H adsorption on the CeO_2−δ_ and M_x_Ce_1−x_O_2−δ_ surface, the free energy of CeO_2−δ_ and M_x_Ce_1−x_O_2−δ_ surface, and the free energy of H_2_ in a rectangular periodic box of size 24.6× 24.6 × 24.0 Å and a 1 × 1 × 1 Monkhorst-Pack k-point grid for Brillouin zone sampling, respectively. The energy barrier for the decomposition of H_2_O was determined by a combination of the nudged elastic band (NEB) method ^[10−13]^. In the NEB method, the chemical reaction path between the two adsorptions (H_2_O* and H−OH*) sites is discretized into a series of structural images.

***Supplementary Figures:***


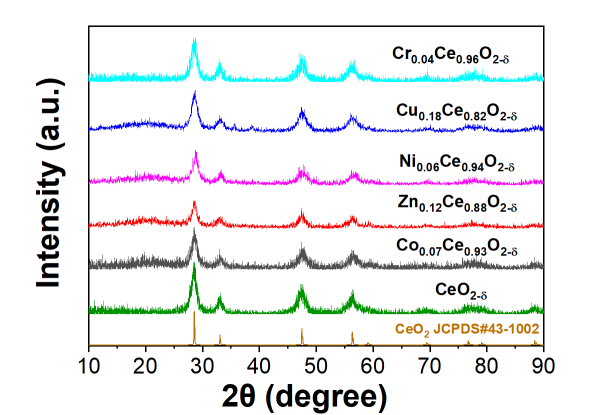


**Figure S1**. XRD patterns of M_x_Ce_1−x_O_2−δ._


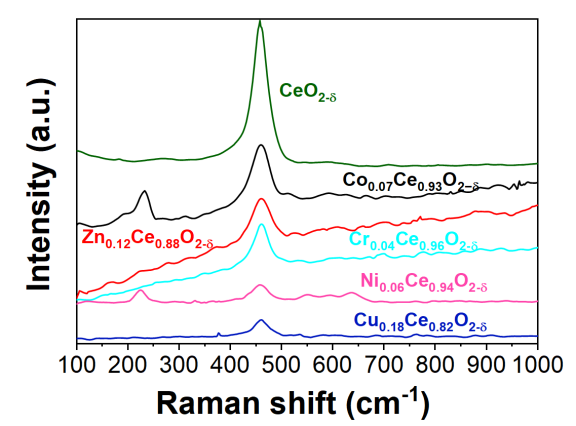


**Figure S2**. Raman spectra of M_x_Ce_1−x_O_2−δ._


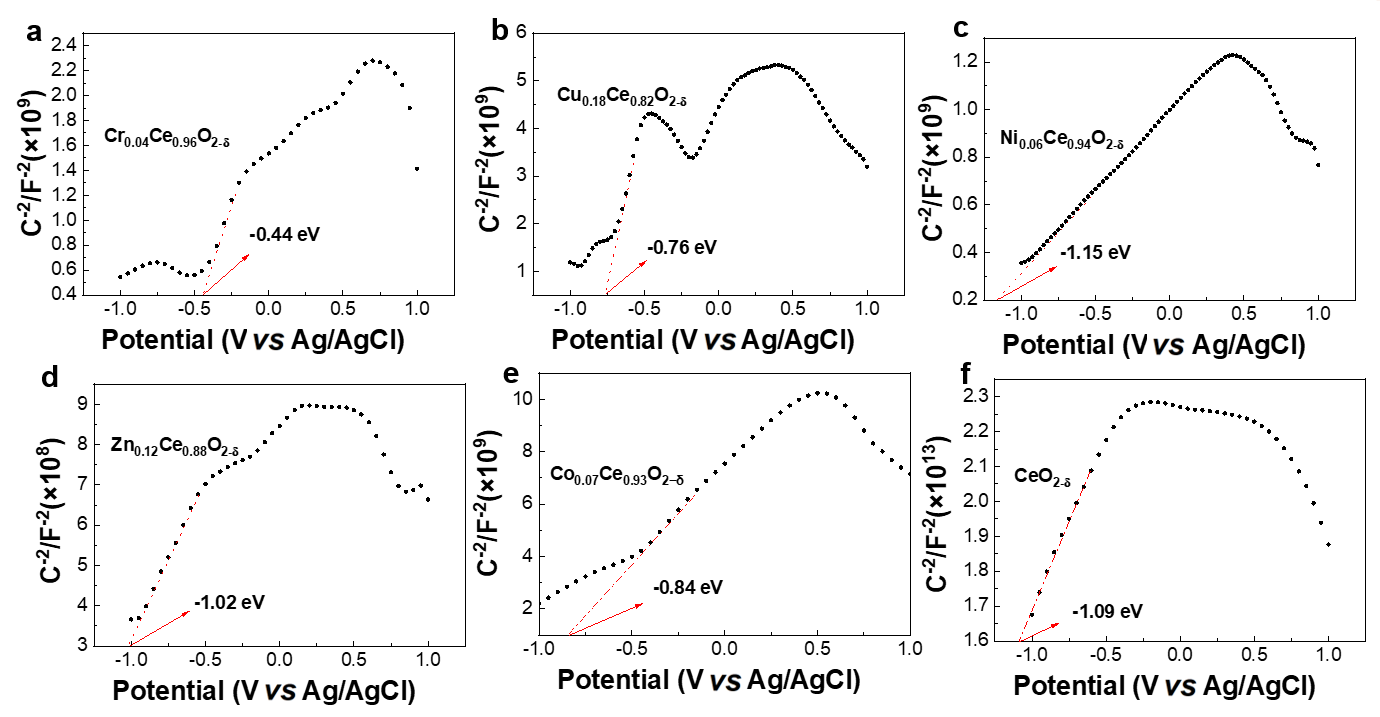


**Figure S3**. Mott Schottky curve of M_x_Ce_1−x_O_2−δ_.


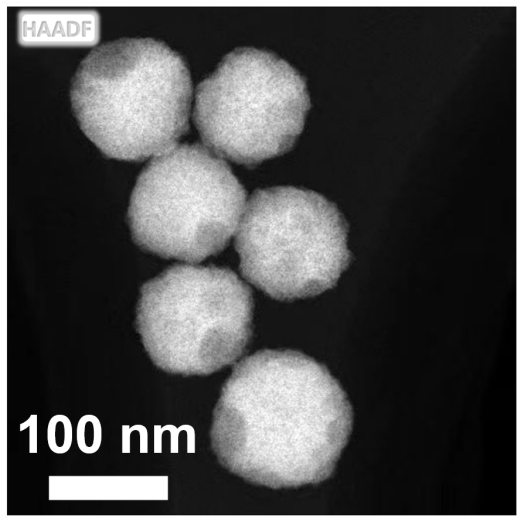


**Figure S4**. HAADF-STEM images of CeO_2−δ._


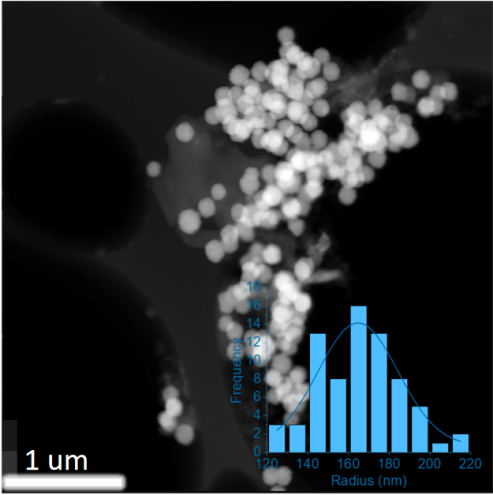


**Figure S5**. Transmission electron micrograph (TEM) image of Co_0.07_Ce_0.93_O_2−δ_ along with a histogram of the diameters.


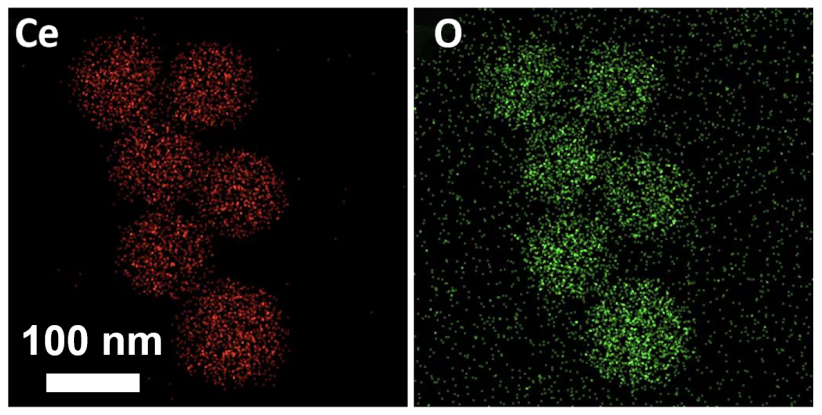


**Figure S6**. EDX elemental mappings of CeO_2−δ_.


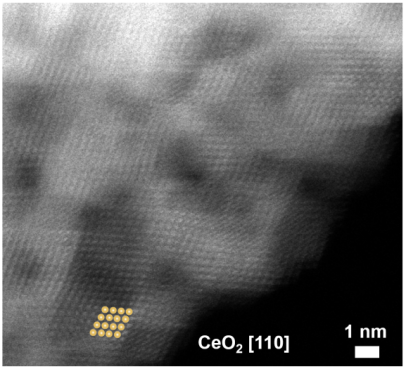


**Figure S7**. HR-STEM images of CeO_2−δ_.


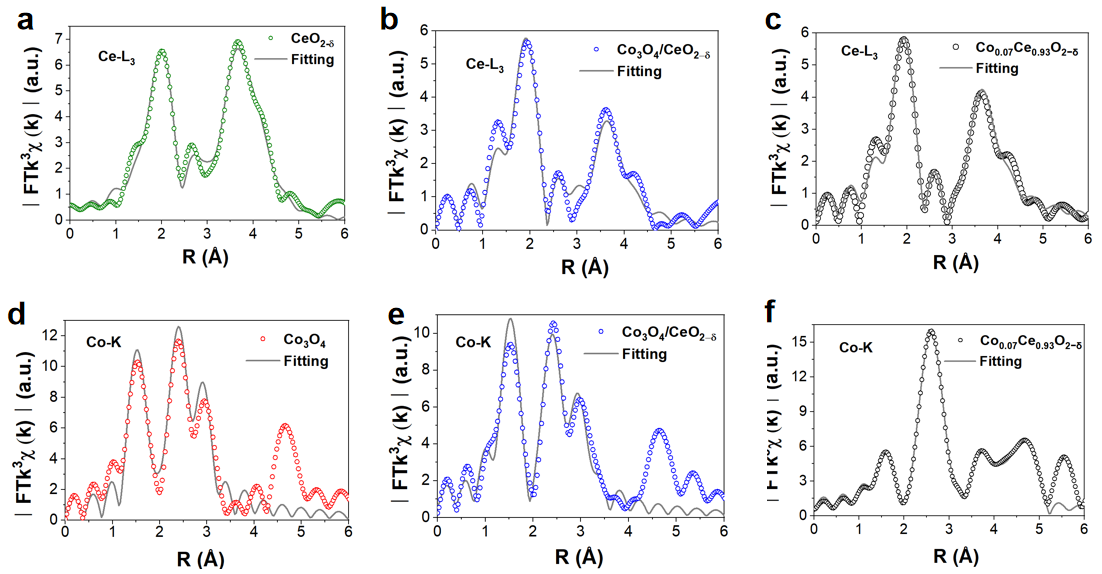


**Figure S8**. The fitting results of Ce L_3_-edge and Co K-edge EXAFS spectra in R-space. In the figures, the solid black line represents the fitting data, while the colored circular lines represent the actual data.


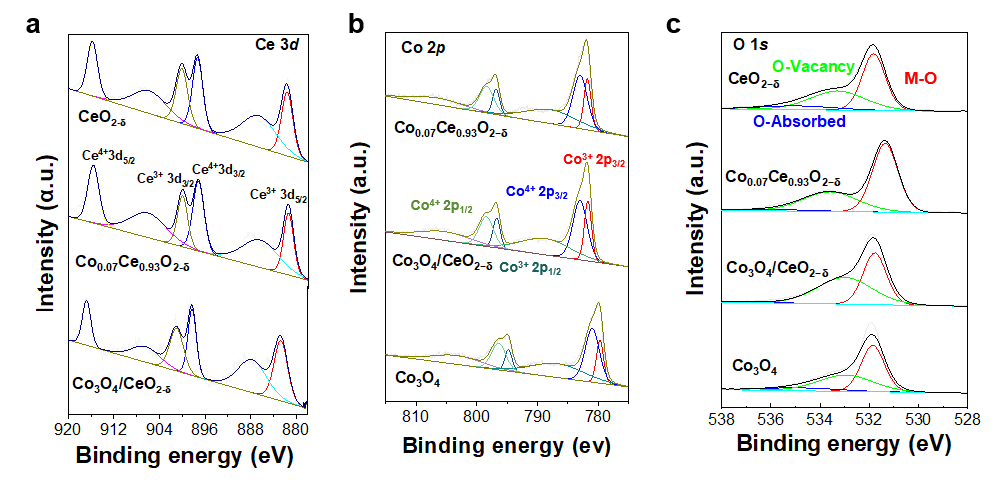


**Figure S9**. XPS of (a) Ce 3*d*, (b) Co 2*p* and (c) O 1*s* for CeO_2−δ_, Co_0.07_Ce_0.93_O_2−δ_, Co_3_O_4_/CeO_2−δ_ and Co_3_O_4_.


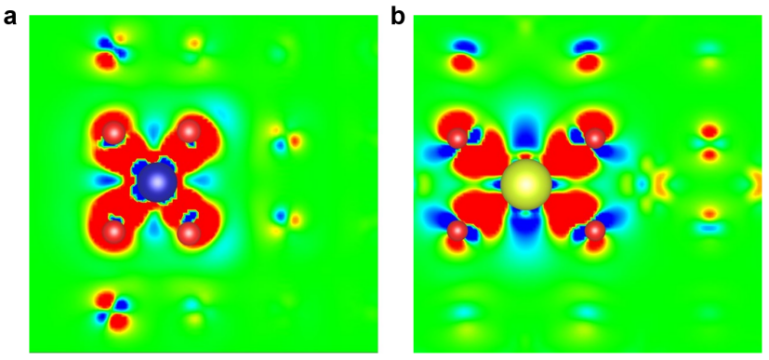


**Figure S10**. Two-dimensional differential charge of (a) CeO_2−δ_ and (b) Co_0.07_Ce_0.93_O_2−δ_.


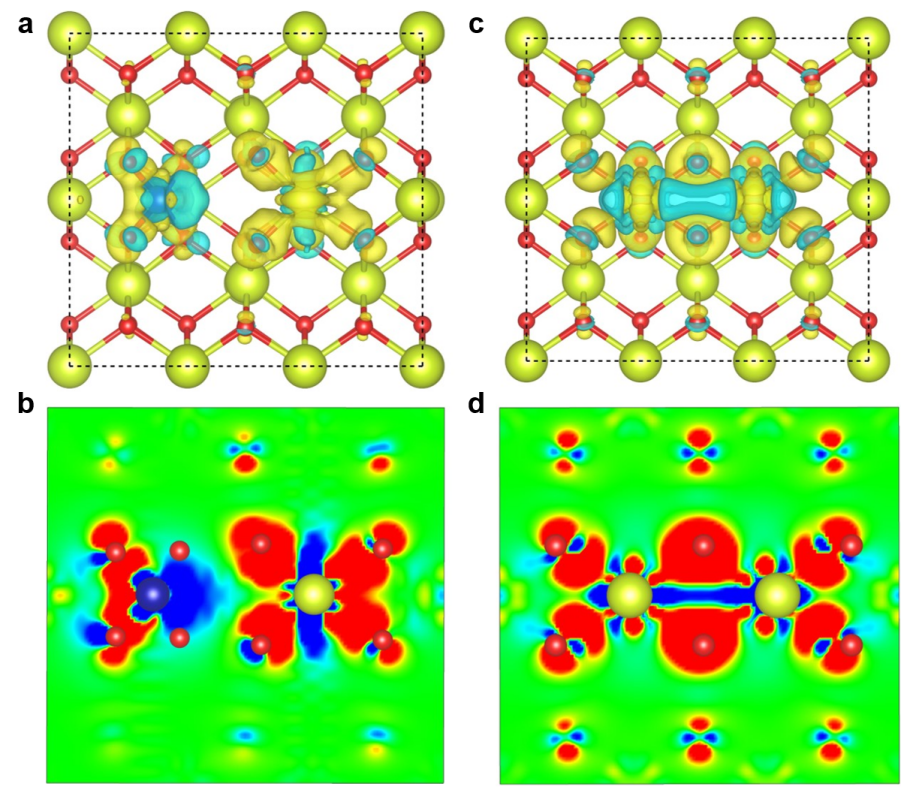


**Figure S11**. The differential charge and two-dimensional differential charge of the two atoms (a, b) CeO_2−δ_ and (c, d) Co_0.07_Ce_0.93_O_2−δ_.


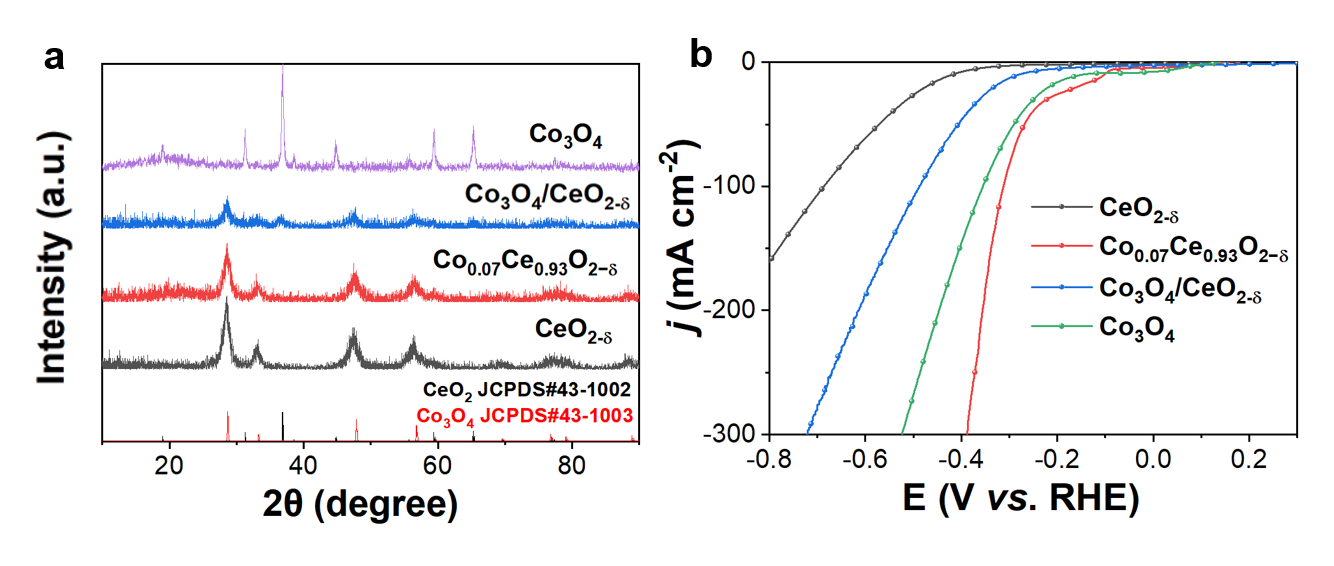


**Figure S12**. (a) XRD patterns and (b) LSV polarization curves of CeO_2−δ_, Co_0.07_Ce_0.93_O_2−δ_, Co_3_O_4_/CeO_2−δ_ and Co_3_O_4_.


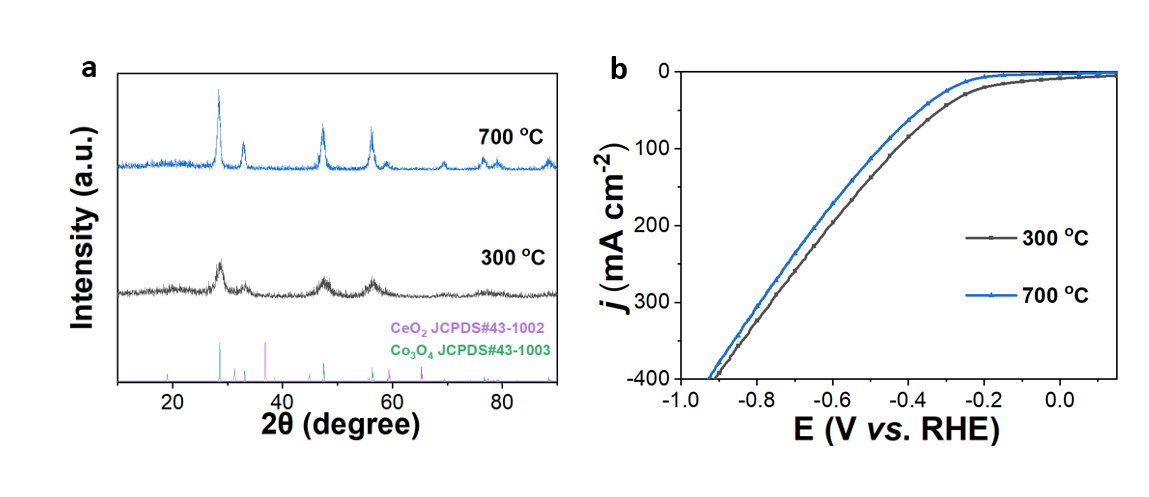


**Figure S13**. (a) XRD patterns and (b) LSV polarization curves of Co_0.07_Ce_0.93_O_2−δ_ solid solution materials annealing under 300 ^o^C and 700 ^o^C.


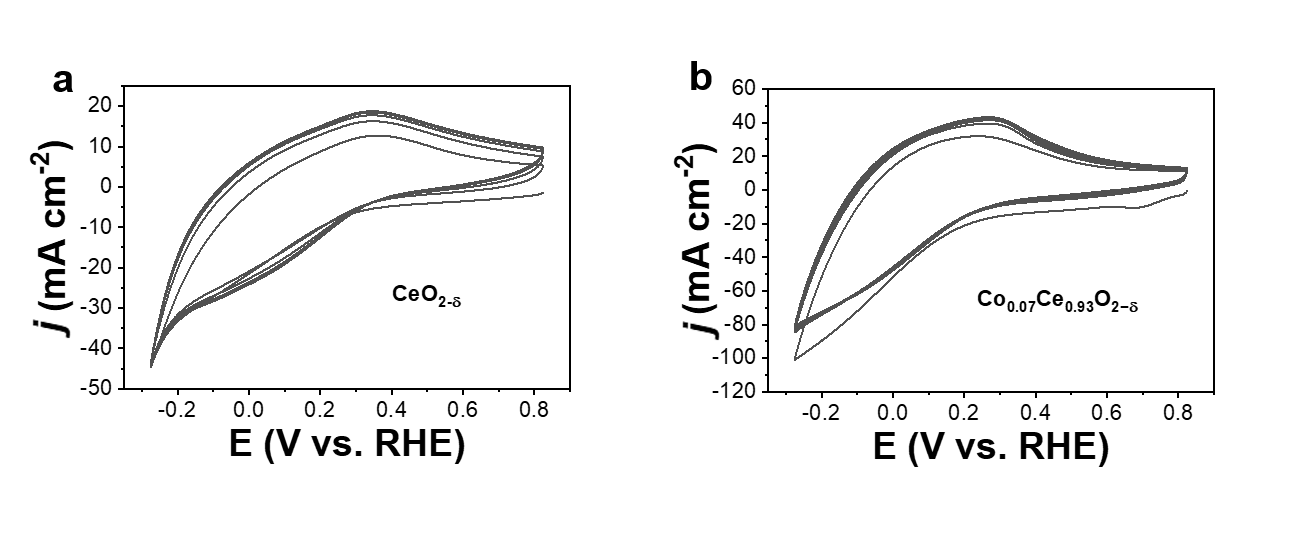


**Figure S14**. The CV polarization curves of CeO_2−δ_ and Co_0.07_Ce_0.93_O_2−δ_.


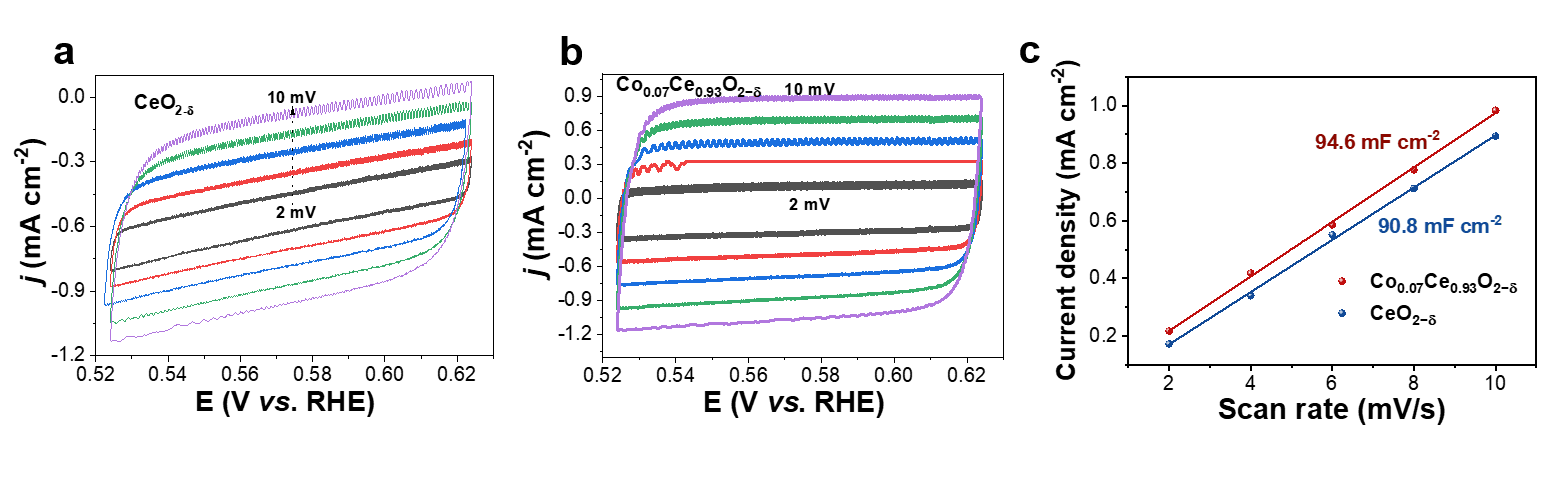


**Figure S15**. The CDL of CeO_2−δ_ and Co_0.07_Ce_0.93_O_2−δ_.


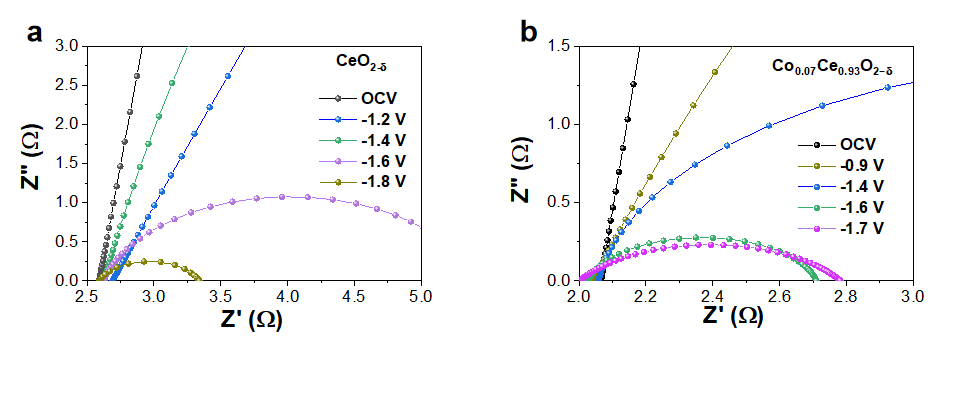


**Figure S16**. The EIS of CeO_2−δ_ and Co_0.07_Ce_0.93_O_2−δ_.


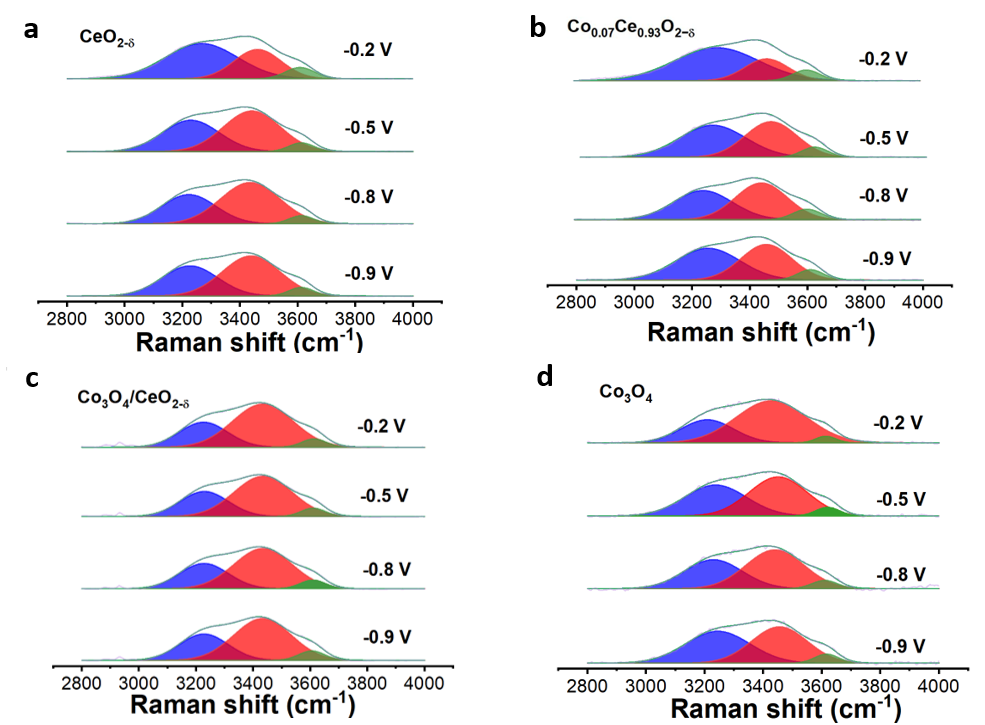


**Figure S17**. In-situ Raman spectra of interfacial water fitting results for (a) CeO_2−δ_, (b) Co_0.07_Ce_0.93_O_2−δ_, (c) Co_3_O_4_/CeO_2−δ_ and (d) Co_3_O_4_ of 4-HB**^.^**H_2_O, 2-HB**^.^**H_2_O and K^+^**^.^**H_2_O are shown in blue, red and green, respectively.


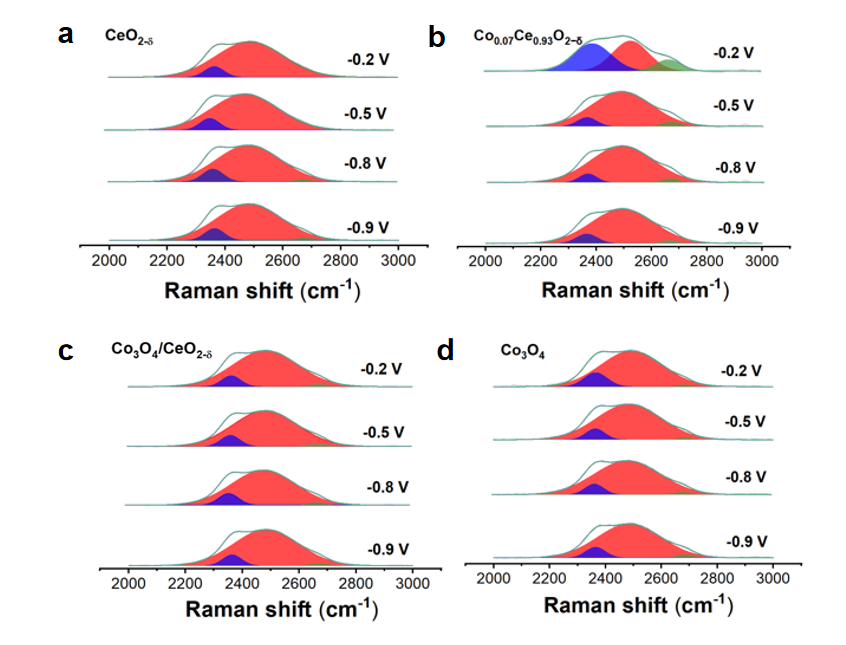


**Figure S18**. In situ Raman spectra of interfacial water fitting results for (a) CeO_2−δ_, (b) Co_0.07_Ce_0.93_O_2−δ_, (c) Co_3_O_4_/CeO_2−δ_ and (d) Co_3_O_4_ of 4-HB**^.^**H_2_D, 2-HB**^.^**H_2_D and K^+^**^.^**H_2_D are shown in blue, red and green, respectively.


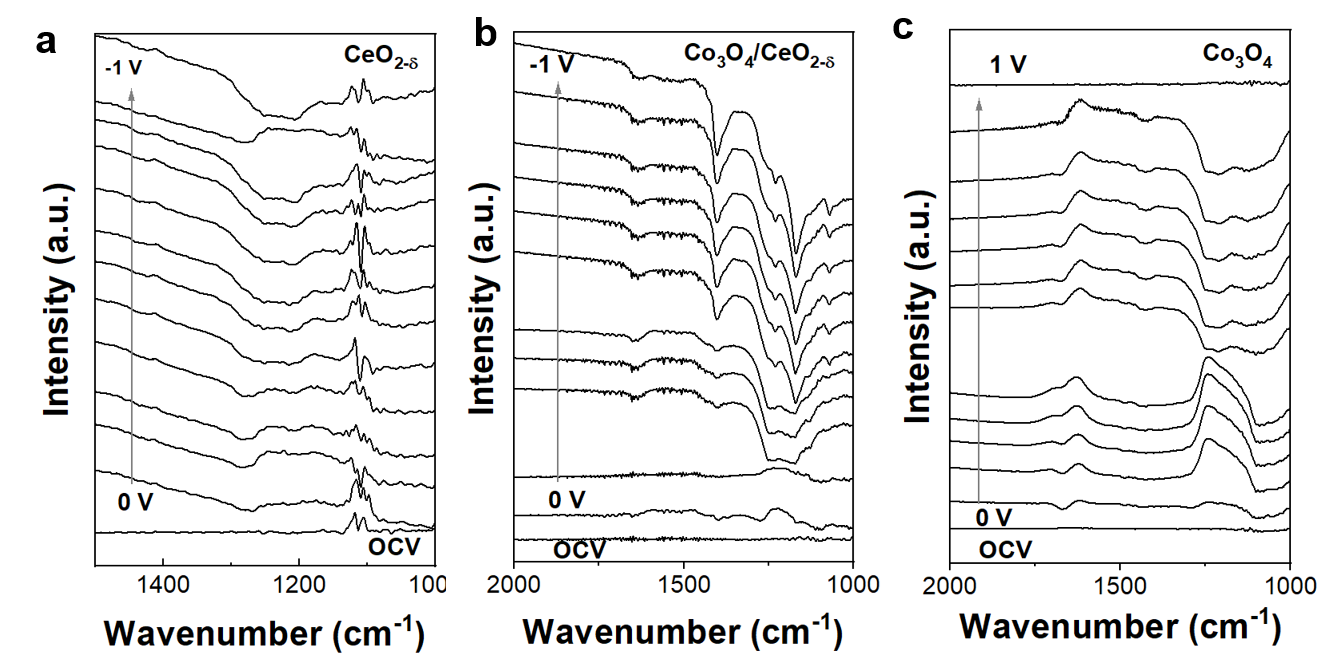


**Figure S19**. In-situ ATR-IR spectra of (a) CeO_2−δ_, (b) Co_3_O_4_/CeO_2−δ_ and (c) Co_3_O_4_.


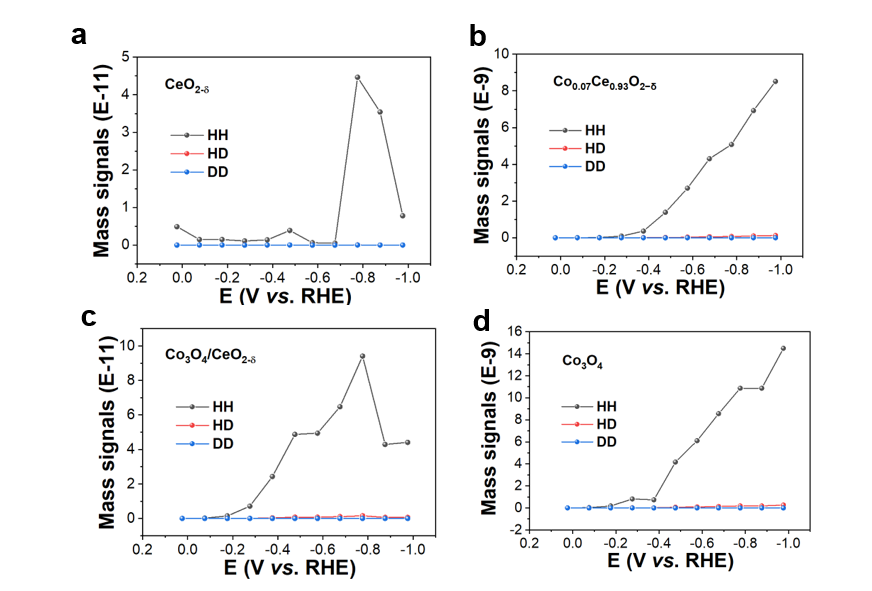


**Figure S20**. The DEMS signals of (a) CeO_2−δ_, (b) Co_0.07_Ce_0.93_O_2−δ_, (c) Co_3_O_4_/CeO_2−δ_ and (d) Co_3_O_4_ were measured at m/z = 2, 3, and 4 in a 1.0 M KOH-H_2_O solution.


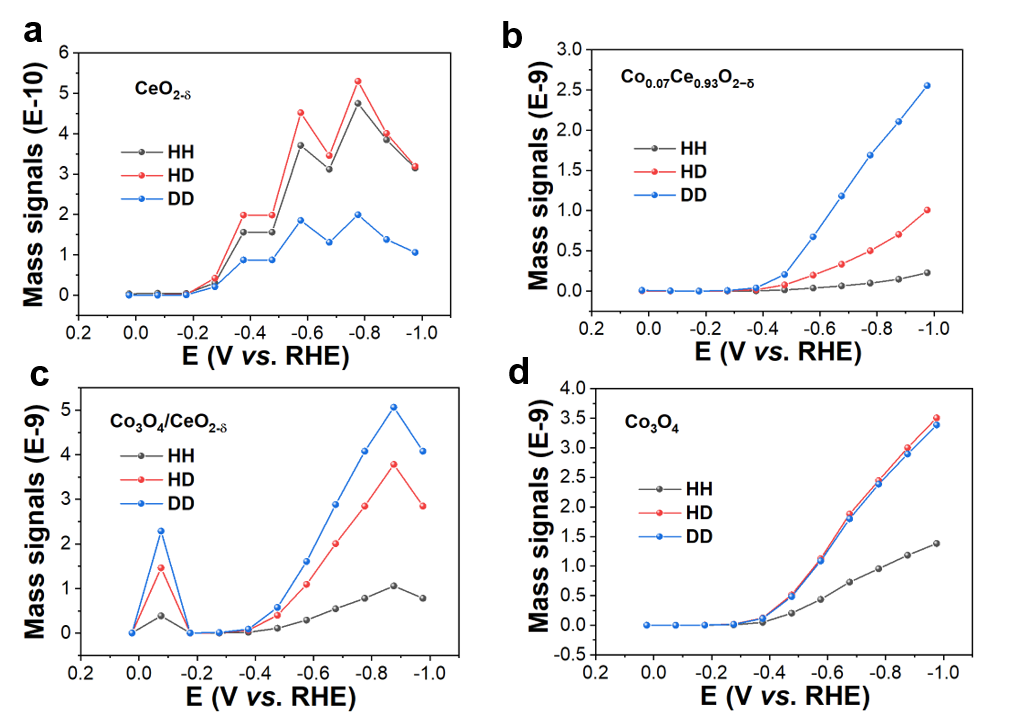


**Figure S21**. The DEMS signals of (a) CeO_2−δ_, (b) Co_0.07_Ce_0.93_O_2−δ_, (c) Co_3_O_4_/CeO_2−δ_ and (d) Co_3_O_4_ were measured at m/z = 2, 3, and 4 in a 1.0 M KOH-D_2_O solution.


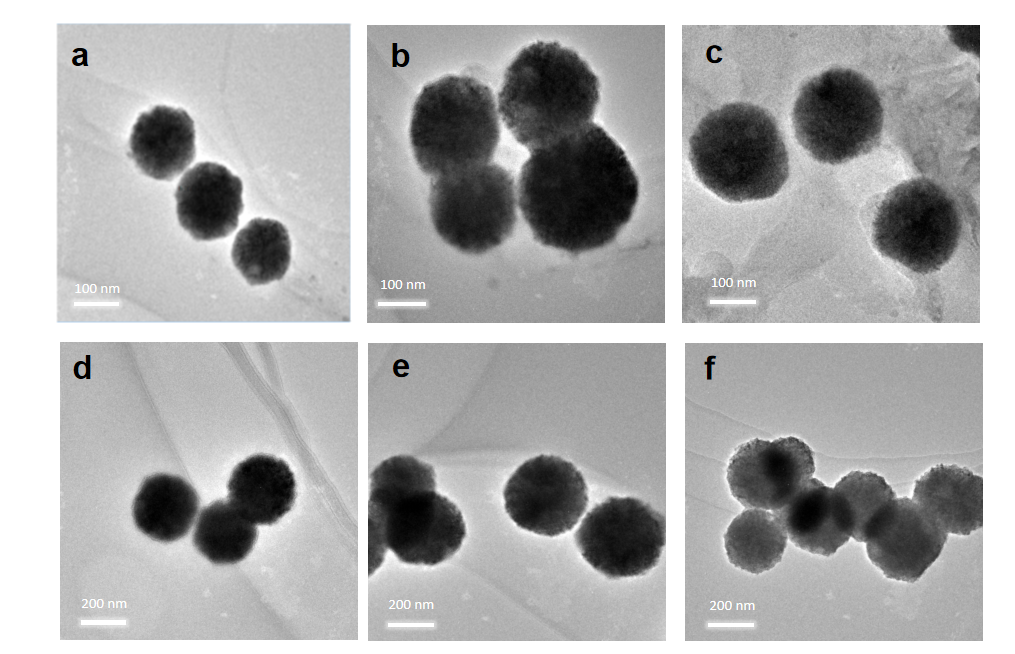


**Figure S22**. TEM image of Co_0.07_Ce_0.93_O_2__−δ_ before (a−c) and after (d−f) HER tests.


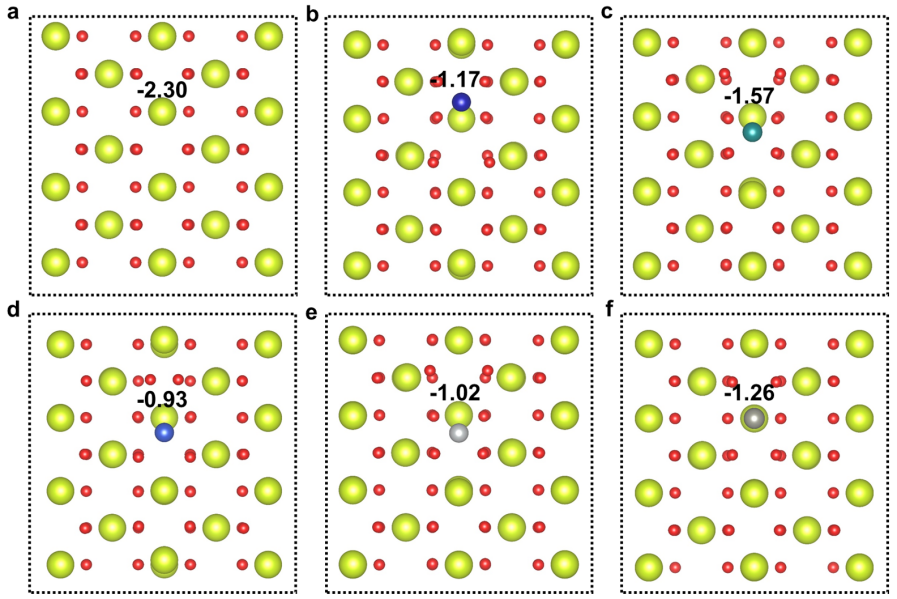


**Figure S23**. The Bader net charge value of (a) CeO_2−δ_, and (b) Co, (c) Cr, (d) Cu, (e) Ni, (f) and Zn substituted CeO_2−δ_ solid solution.


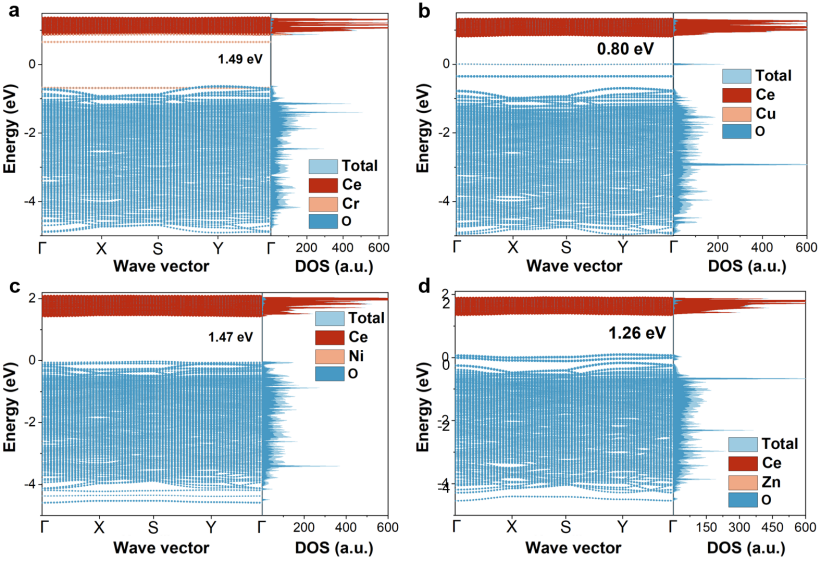


**Figure S24**. The DOS and Band of (a) Cr, (b) Cu, (c) Ni and (d) Zn substituted CeO_2−δ_ solid solution.


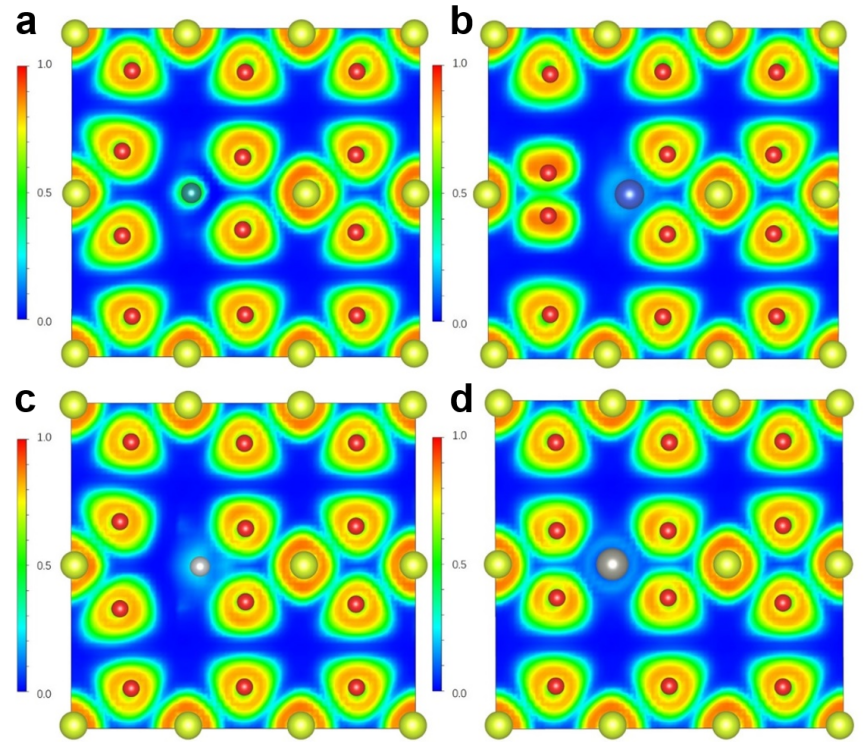


**Figure S25**. The ELF of (a) Cr, (b) Cu, (c) Ni and (d) Zn substituted CeO_2−δ_ solid solution.


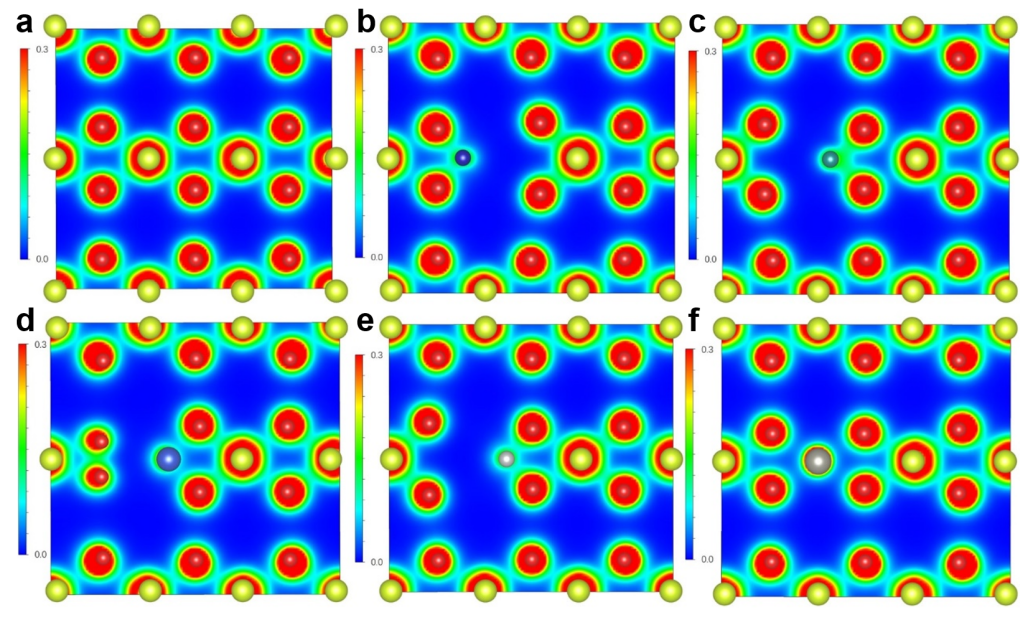


**Figure S26**. 2D-charge density cross section of (a) CeO_2−δ_, and (b) Co, (c) Cr, (d) Cu, (e) Ni, (f) Zn substituted CeO_2−δ_ solid solution.


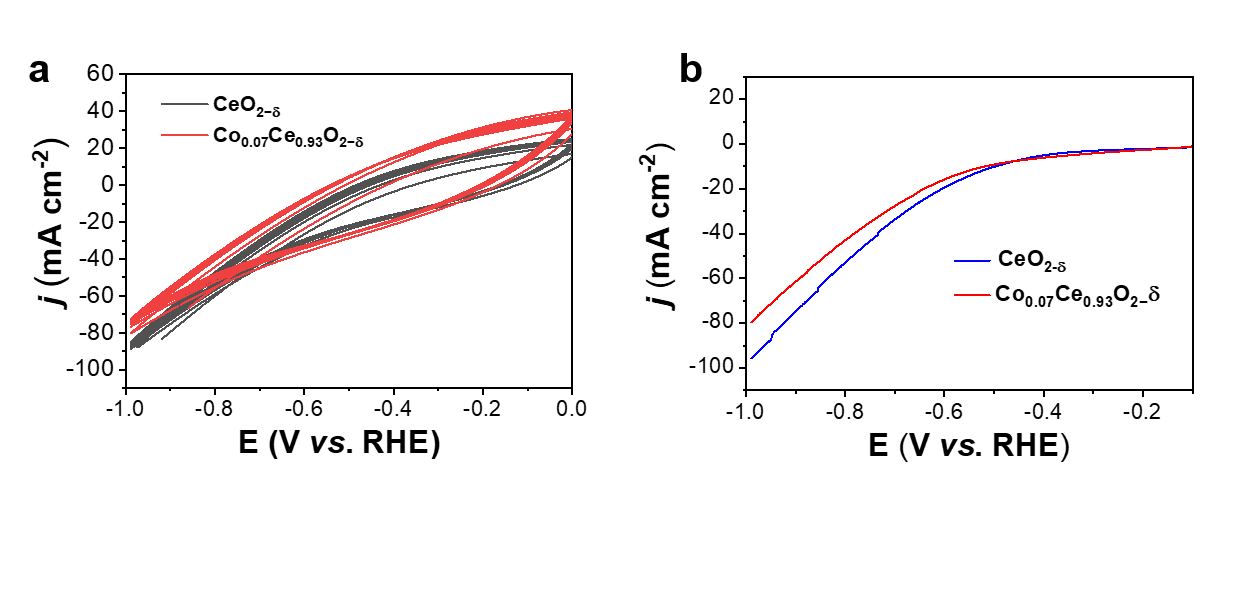


**Figure S27**. The CV and LSV of CeO_2−δ_ and Co_0.07_Ce_0.93_O_2−δ_ in actual seawater.


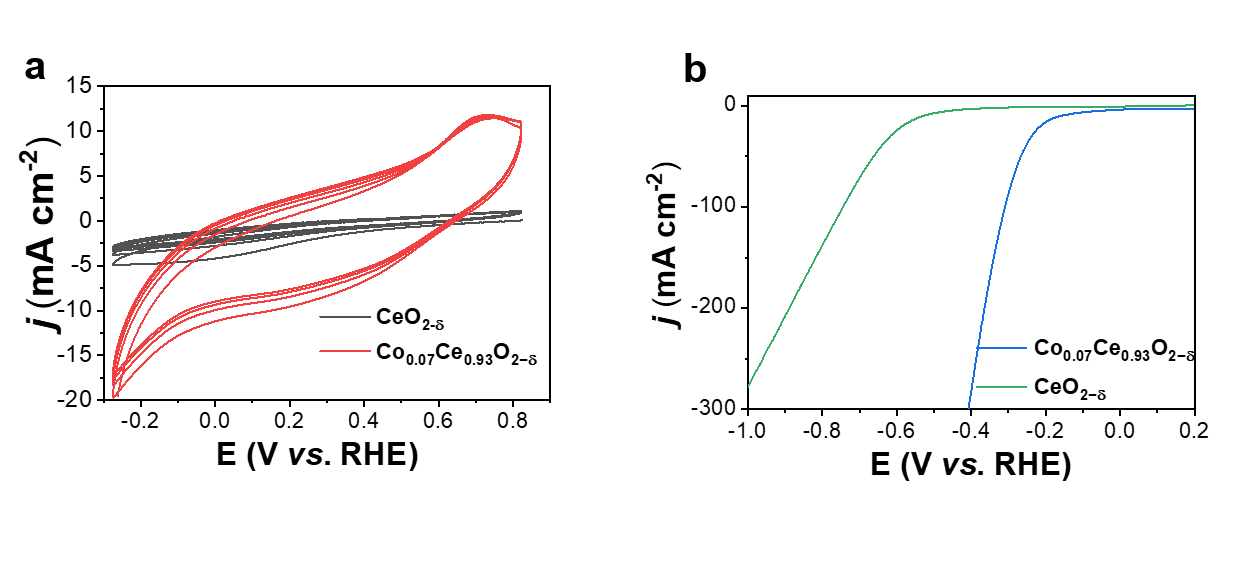


**Figure S28**. The CV and LSV of CeO_2−δ_ and Co_0.07_Ce_0.93_O_2−δ_ in 1.0 M alkaline seawater.


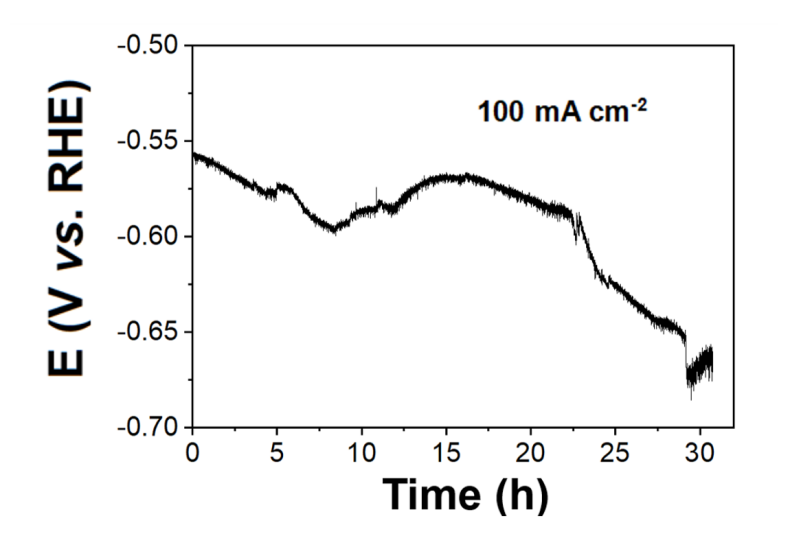


**Figure S29**. The stability testing of Pt/C (20%) in 1.0 M alkaline seawater.

***Supplementary Tables:***

**Table S1.** ICP-OES measurements for M_x_Ce_y_O_2−δ._

| Catalysts | Ce wt% (mol L^-1^) | M wt% (mol L^-1^) |
| --- | --- | --- |
| Cu_0.18_Ce_0.82_O_2−δ_ | 0.040 | 0.008 |
| Co_0.07_Ce_0.93_O_2−δ_ | 0.049 | 0.02 |
| Cr_0.04_Ce_0.96_O_2−δ_ | 0.047 | 0.00012 |
| Ni_0.06_Ce_0.94_O_2−δ_ | 0.046 | 0.003 |
| Zn_0.12_Ce_0.88_O_2−δ_ | 0.048 | 0.006 |

**Table S2.** The bandgap widths of the conduction and valence bands for material M_x_Ce_y_O_2-δ._

| Catalysts | E_g_ (eV) | E_CB_ (*vs*. NHE) | E_VB_ (*vs*. NHE) |
| --- | --- | --- | --- |
| Cu_0.18_Ce_0.82_O_2−δ_ | 3.22 | -0.76 | 2.46 |
| Co_0.07_Ce_0.93_O_2−δ_ | 3.08 | -0.84 | 2.24 |
| Cr_0.04_Ce_0.96_O_2−δ_ | 3.28 | -0.44 | 2.84 |
| Ni_0.06_Ce_0.94_O_2−δ_ | 3.03 | -1.15 | 1.88 |
| Zn_0.12_Ce_0.88_O_2−δ_ | 2.93 | -1.02 | 1.91 |
| CeO_2−δ_ | 3.12 | -1.11 | 2.01 |

**Table S3.** Fitting parameters and results of the Ce 3*d* spectrum in Figure S9.

| catalysts  peak | CeO_2−δ_ | | Co_0.07_Ce_0.93_O_2−δ_ | | Co_3_O_4_/CeO_2−δ_ | |
| --- | --- | --- | --- | --- | --- | --- |
|  | binding energy | half-peak width | binding energy | half-peak width | binding energy | half-peak width |
| Ce^3+^ 3*d*_5/2_ | 882.53 | 1.84 | 882.33 | 1.84 | 882.54 | 1.84 |
| Ce^4+^ 3*d*_5/2_ | 916.86 | 2.40 | 916.66 | 2.40 | 916.80 | 2.40 |
| Ce^3+^ 3*d*_3/2_ | 901.01 | 2.40 | 900.88 | 2.40 | 901.08 | 2.40 |
| Ce^4+^ 3*d*_3/2_ | 898.41 | 1.84 | 898.20 | 1.84 | 898.33 | 1.84 |

**Table S4.** Fitting parameters and results of the Co 2*p* spectrum in Figure S9.

| catalysts  peak | Co_0.07_Ce_0.93_O_2−δ_ | | Co_3_O_4_/CeO_2−δ_ | | Co_3_O_4_ | |
| --- | --- | --- | --- | --- | --- | --- |
|  | binding energy | half-peak width | binding energy | half-peak width | binding energy | half-peak width |
| Co^3+^ 2*p*_3/2_ | 781.77 | 1.38 | 781.70 | 1.38 | 779.72 | 1.38 |
| Co^2+^ 2*p*_3/2_ | 782.98 | 2.77 | 783.00 | 2.77 | 780.98 | 2.77 |
| Co^3+^ 2*p*_1/2_ | 796.81 | 1.38 | 796.73 | 1.38 | 794.76 | 1.38 |
| Co^2+^ 2*p*_1/2_ | 798.36 | 2.77 | 798.41 | 2.77 | 796.40 | 2.77 |

**Table S5.** Fitting parameters and results of the O 1*s* spectrum in Figure S9.

| catalysts  peak | CeO_2−δ_ |  | Co_0.07_Ce_0.93_O_2−δ_ | | Co_3_O_4_/CeO_2−δ_ | | Co_3_O_4_ | |
| --- | --- | --- | --- | --- | --- | --- | --- | --- |
|  | binding energy | half-peak width | binding energy | half-peak width | binding energy | half-peak width | binding energy | half-peak width |
| O-Vacancy | 533.28 | 2.62 | 533.73 | 2.62 | 533.02 | 2.62 | 532.96 | 2.62 |
| O-Absorbed | 534.83 | 3.74 | 534.47 | 3.74 | 534.22 | 3.74 | 534.77 | 3.74 |
| M-O | 531.82 | 1.11 | 531.35 | 1.11 | 531.90 | 1.11 | 531.86 | 1.11 |

**Table S6.** Parameters from the refinement of Ce L_3_-edge and Co K-edge EXAFS fitting.

| Catalysts | Bond | Coordination number | R (Å) | σ^2^ × 10^3^ (Å^2^) | R-factor |
| --- | --- | --- | --- | --- | --- |
| CeO_2−δ_ | Ce−O | 7.80 | 2.58 | 4.19 ± 0.83 | 0.0040 |
|  | Ce−O−Ce | 10.10 | 3.76 | 3.92 ± 0.65 |  |
| Co_3_O_4_/CeO_2−δ_ | Ce−O | 7.60 | 2.56 | 3.68 ± 0.53 | 0.0060 |
|  | Ce−O−Ce | 9.60 | 3.81 | 4.12 ± 0.93 |  |
| Co_0.07_Ce_0.93_O_2−δ_ | Ce−O | 6.80 | 2.58 | 5.32 ± 0.68 | 0.0030 |
|  | Ce−O−Ce | 11.20 | 3.99 | 5.03 ± 0.76 |  |
| Co_3_O_4_ | Co−O | 5.80 | 1.72 | 2.19 ± 0.43 | 0.0050 |
|  | Co−O−Co | 6.00 | 2.68 | 2.65 ± 0.21 |  |
| Co_3_O_4_/CeO_2−δ_ | Co−O | 5.60 | 1.94 | 3.19 ± 0.53 | 0.0030 |
|  | Co−O−Co | 5.40 | 2.76 | 3.52 ± 0.33 |  |
| Co_0.07_Ce_0.93_O_2−δ_ | Co−O | 4.20 | 1.82 | 3.79 ± 0.83 | 0.0060 |
|  | Co−O−Co | 4.80 | 2.70 | 3.19 ± 0.43 |  |

**Table S7.** HER performance compared between Co_0.07_Ce_0.93_O_2−δ_ and other reported catalysts in 1.0 M KOH.

| Catalysts | *η* @ *j* (mV) | Stability | Reference |
| --- | --- | --- | --- |
| Co_0.07_Ce_0.93_O_2−δ_ | 75 @ 10 mA cm^−2^ | 300 h @ 10, 50 and 100 mA cm^−2^ | This work |
| W_1_N_1_C_3_ | 85 @ 10 mA cm^−2^ | − | [1] |
| CoSe@NCNSs | 170@ 10 mA cm^−2^ | 10 h @ 200 mA cm^−2^ | [2] |
| 1T_0.72_-MoS_2_@NiS_2_ | 95@ 10 mA cm^−2^ | − | [3] |
| Co-1T-MoS_2_-bpe-350 | 118@ 10 mA cm^−2^ | 10 h @ 60 mA cm^−2^ | [4] |
| CoSe_2_/Co_3_S_4_@Co_3_O_4_ | 165@ 10 mA cm^−2^ | − | [5] |
| CoNC‑SA/N*−C | 194@ 10 mA cm^−2^ | 50 h @ 12 mA cm^−2^ | [6] |
| Ru/Mo_2_N | 66@ 10 mA cm^−2^ | − | [7] |
| Co_3_S_4_/MoS_2_/Ni_2_P | 178@ 10 mA cm^−2^ | − | [8] |
| ZnCo_2_O_4_ | 401@ 10 mA cm^−2^ | − | [9] |
| Ir-Co_3_O_4_@NC  Ce-CoP@CC  Co_9_S_8_-MoS_2_/NF  Co−MoS_2_ | 188@ 10 mA cm^−2^  81@ 10 mA cm^−2^  110@ 10 mA cm^−2^  105@ 10 mA cm^−2^ | 12 h @ 10 mA cm^−2^  25h @ 10 mA cm^−2^  −  25h @ 10 mA cm^−2^ | [10]  [11]  [12]  [13] |

**Table S8.** HER performance compared between Co_0.07_Ce_0.93_O_2−δ_ and other reported catalysts in alkaline seawater.

| Catalysts | *η* @ *j* (mV) | Stability | Reference |
| --- | --- | --- | --- |
| Co_0.07_Ce_0.93_O_2−δ_ | 160 @ 10 mA cm^−2^ | 300 h @ 10, 50 and 100 mA cm^−2^ | This work |
|  | 309 @ 100 mA cm^−2^ |  |  |
|  | 474 @ 500 mA cm^−2^ |  |  |
| NiPS/NF | 136.2 @ 10 mA cm^−2^  329 @ 100 mA cm ^−2^ | 24 h @10mA cm ^−2^  60 h @ 200 mA cm ^−2^ | [14] |
| Co/Co_9_S_8_ | 194 @ 20 mA cm^−2^ | − | [15] |
| Co- Ni-S/NF | 236 @ 50 mA cm^−2^ | − | [16] |
| (Co,Fe)PO_4_ | 136 @ 10 mA cm^−2^ | 70h @ 100 mA cm^−2^ | [17] |
| Co-P-B/NF | 170 @ 10 mA cm^−2^ | − | [18] |
| PdCo-Co_3_S_4_ | 163@ 10 mA cm^−2^ | 100h @ 10mA cm^−2^ | [19] |
| Co_x_P_y_/Ni_x_P_y_-NPC | 203@ 10 mA cm^−2^ | 10 h @ 15.3 mA mg^−1^ | [20] |
| Ru, W–NiSe_2_/NF | 353@ 10 mA cm^−2^ | − | [21] |

***Supplementary references:***

[1] Kresse, G.; Furthmüller, J. Efficiency of Ab-Initio Total Energy Calculations for Metals and Semiconductors Using a Plane-Wave Basis Set. *Comput. Mater. Sci*. **1996**, *6*, 15–50.

[2] Kresse, G; J Furthmüller. Efficient iterative schemes for ab initio total-energy calculations using a plane-wave basis set. *Physical review. B Condens matter*. **1996**, *54*, 11169–11186.

[3] Li, W.; Wang, F.; Liu, X.; Dang, Y.; Li, J.; Ma, T.; Wang, C. Promoting Body Carriers Migration of CdS Nanocatalyst by N-Doping for Improved Hydrogen Production under Simulated Sunlight Irradiation. *Appl. Catal. B Environ.* **2022**, *313*, 121470.

[4] Perdew, J. P.; Burke, K.; Ernzerhof, M. Generalized Gradient Approximation Made Simple. *Phys. Rev. Lett.* **1996**, *77*, 3865–3868.

[5] Kresse, G.; Joubert, D. From Ultrasoft Pseudopotentials to the Projector Augmented-Wave Method. *Phys. Rev. B* **1999**, *59*, 1758–1775.

[6] Engel, M.; Marsman, M.; Franchini, C.; Kresse, G. Electron-Phonon Interactions Using the Projector Augmented-Wave Method and Wannier Functions. *Phys. Rev. B* **2020**, *101*, 184302.

[7] Liu, X.; Cao, S.; Li, J.; Wang, Y.; Xue, W.; Liu, G. Protective Cerium Oxide Coating Promoted Ce-Doping and Reconstruction of High-Valence NiFe Sulfide toward Robust Overall Water Splitting. *Small* **2023**, *19*, 2304652.

[8] Liu, X.; Wei, S.; Cao, S. Lattice Strain with Stabilized Oxygen Vacancies Boosts Ceria for Robust Alkaline Hydrogen Evolution Outperforming Benchmark Pt. *Adv. Mater*. **2024**, DOI: 10.1002/adma.202405970.

[9] Wang, Y.; Qiao, M.; Li, Y.; Wang, S. Tuning Surface Electronic Configuration of NiFe LDHs Nanosheets by Introducing Cation Vacancies (Fe or Ni) as Highly Efficient Electrocatalysts for Oxygen Evolution Reaction. *Small* **2018**, *14*, 1800136.

[10] Chen, S.; Li, S.; You, R.; Guo, Z.; Wang, F.; Li, G.; Yuan, W.; Zhu, B.; Gao, Y.; Zhang, Z.; Yang, H.; Wang, Y. Elucidation of Active Sites for CH_4_ Catalytic Oxidation over Pd/CeO_2_ Via Tailoring Metal–Support Interactions. *ACS Catal.* **2021**, *11*, 5666–5677.

[11] Ricciardulli, T.; Gorthy, S.; Adams, J. S.; Thompson, C.; Karim, A. M.; Neurock, M.; Flaherty, D. W. Effect of Pd Coordination and Isolation on the Catalytic Reduction of O_2_ to H_2_O_2_ over PdAu Bimetallic Nanoparticles. *J. Am. Chem. Soc.* **2021**, *143*, 5445–5464.

[12] Patel, V. R.; Somaiya, R. N.; Kansara, S.; Singh, D.; Prajapati, N.; Sonvane, Y.; Thakor, P. B.; Structural and electrical properties of CeO_2_ Solid State Communications. *State Commun*. **2020**, *307*, 113801.

[13] Chen, Z.-G.; Xu, Y.-F.; Ding, D.; Song, G.; Gan, X.-X.; Zhao, Z.-G.; Cui, Y. Thermal migration towards constructing W-W dual-sites for boosted alkaline hydrogen evolution reaction. *Nat. Commun*. **2022**, *9*, 13763.

[14] Sun, C.-C.; Wang, C.; Xie, H.-J.; Han. G.T.; Zhang, Y.-M.; Zhao, H.-G. 2D cobalt chalcogenide heteronano structures enable efficient alkaline hydrogen evolution reaction. *Small* **2023**, *19*, 2302056.

1. Liu, M.; Wang, J. A.; Klysubun W. et al. Interfacial electronic structure engineering on molybdenum sulfide for robust dual-pH hydrogen evolution. *Nat Commun*. **2021**, *12*, 5260.
2. Liu, H.-J.; Zhang, S.; Chai, Y.-M; Dong, B. Ligand Modulation of Active Sites to Promote Cobalt-Doped 1T-MoS_2_ Electrocatalytic Hydrogen Evolution in Alkaline Media. *Angew. Chem., Int. Ed*. **2023**, *62*, e202313845.
3. Wang, C.; Du, X.; Zhang, X. Controlled Synthesis of W–Co3S4@Co3O4 as an Environmentally Friendly and Low Cost Electrocatalyst for Overall Water Splitting. *Int. J. Hydro. Energy*. **2023**, *48*, 12752.
4. Wang, M.- M.; Sun, K. A.; Mi, W. L.; Feng, C.; Guan, Z. K.; Liu, Y. Q.; Pan, Y. *ACS Catal*. **2022**, *12*, 10771–10780.
5. Hou, X.; Yu, X.; Liu, M.; Peng, H.; Wu, L.; Liao, L.; Lv, G. Ultrafast Synthesis of Mo_2_N with Highly Dispersed Ru for Efficient Alkaline Hydrogen Evolution. *Chin. Chem. Lett.* **2024**, DOI: 10.1016/j.cclet.2024.109845.

[20] Ou, H.-H.; Lin, L.-H.; Zheng, Y.; Yang, P.-J.; Fang, Y.-X.; Wang, X.-C. Tri‐s‐triazine‐Based Crystalline Carbon Nitride Nanosheets for an Improved Hydrogen Evolution. *Adv. Mater*. **2017**, *29*, 1700008.

[21] Wu, J.; Wang, X.; Zheng, W.; Sun, Y.; Xie, Y.; Ma, K.; Zhang, Z.; Liao, Q.; Tian, Z.; Kang, Z.; Zhang, Y. Identifying and Interpreting Geometric Configuration-Dependent Activity of Spinel Catalysts for Water Reduction. *J. Am. Chem. Soc.* **2022**, *144*, 19163–19172.

[22] Jung, S.; Senthil, R. A.; Moon, C. J.; Tarasenka, N.; Min, A.; Lee, S. J.; Tarasenko, N.; Choi, M. Y. Mechanistic Insights into ZIF-67-Derived Ir-Doped Co3O4@N-Doped Carbon Hybrids as Efficient Electrocatalysts for Overall Water Splitting Using in Situ Raman Spectroscopy. *Chem. Eng. J.* **2023**, *468*, 143717.

[23] Li, M.; Wang, X.; Du, H.; Dong, W.; Ye, S.; Liu, H. Oxophilic Tm‐Sites in MoS_2_ Trigger Thermodynamic Spontaneous Water Dissociation for Enhanced Hydrogen Evolution. *Adv. Energy Mater*. **2024**, DOI: 10.1002/aenm.202401716.

[24] Kim, M.; Anjum, M. A. R.; Choi, M.; Jeong, H. Y.; Choi, S. H.; Park, N.; Lee, J. S. Covalent 0D–2D Heterostructuring of Co_9_S_8_–MoS_2_ for Enhanced Hydrogen Evolution in All pH Electrolytes. *Adv. Funct. Mater*. **2020**, *30*, 2002536.

[25] Huang, J.; Hao, M.; Mao, B.; Zheng, L.; Zhu, J.; Cao, M. The Underlying Molecular Mechanism of Fence Engineering to Break the Activity–Stability Trade-Off in Catalysts for the Hydrogen Evolution Reaction. *Angew. Chem. Int. Ed.* **2022**, *61*, e202114899.

[26] Wang, H. Y.; Ren, J. T.; Wang, L.; Sun, M. L.; Yang, H. M.; Lv, X. W.; Yuan, Z. Y. Synergistically enhanced activity and stability of bifunctional nickel phosphide/sulfide heterointerface electrodes for direct alkaline seawater electrolysis. *J. Energy Chem*. **2022**,*75*, 66–73.

[27] Li, J.; Sun, J.; Meng, X. Spherical Co/Co_9_S_8_ as Electrocatalyst for Hydrogen Production from Alkaline Solution and Alkaline Seawater. I*nt. J. Hydro. Energy* **2023**, *48*, 29583–29592.

[28] Gopalakrishnan S.; Saranya V.; Harish S.; et al. Heterogeneous bimetallic oxysulfide nanostructure (Ni-Co) as hybrid bifunctional electrocatalyst for sustainable overall alkaline simulated seawater splitting. *J. Alloys Compd*. **2023**, *965*, 171124.

[29] Kim, C.; Lee, S.; Kim, S. H.; Park, J.; Kim, S.; Kwon, S.-H.; Bae, J.-S.; Park, Y. S.; Kim, Y. Cobalt–Iron–Phosphate Hydrogen Evolution Reaction Electrocatalyst for Solar-Driven Alkaline Seawater Electrolyzer. *Nanomaterials* **2021**, *11*, 2989.

[30] Silviya, R.; Bhide, A.; Gupta, S.; Bhabal, R.; Mali, K. H.; Bhagat, B. R. Bifunctional Amorphous Transition‐Metal Phospho‐Boride Electrocatalysts for Selective Alkaline Seawater Splitting at a Current Density of 2 A cm^−2^. *Small Methods* **2024**, DOI: 10.1002/smtd.202301395.

[31] Sun, P.; Zheng, X.; Chen, A.; Zheng, G.; Wu, Y.; Long, M.; Zhang, Q.; Chen, Y. Constructing Amorphous-Crystalline Interfacial Bifunctional Site Island-Sea Synergy by Morphology Engineering Boosts Alkaline Seawater Hydrogen Evolution. *Adv. Sci.* **2024**, *11*, 2309927.

[32] Zhu, L.; Huang, Y.; Wang, B.; Zhang, Y.; Zou, R.; Yan, L.; Sun, W. N-Doped Porous Carbon-Supported Co_x_P_y_/Ni_x_P_y_ Catalyst with Enhanced Catalytic Activity for Hydrogen Evolution Reaction in Alkaline Solution and Neutral Seawater. *J. Solid State Electrochem.* **2022**, *26*, 233–243.

[33] Dang, Y.; Wang, G.; Li, X.; Ma, X.; Yue, F.; Wang, C.; Gao, L.; Fu, F. Enhanced Alkaline/Seawater Hydrogen Evolution Reaction Performance of NiSe_2_ by Ruthenium and Tungsten Bimetal Doping. *Int. J. Hydro. Energy* **2023**, *48*, 17035.
